# Supplementary material for: Layer-specific, retinotopically-diffuse modulation in human visual cortex in response to viewing emotionally expressive faces
Source: Nat Commun. 2022 Oct 22;13:6302. doi: 10.1038/s41467-022-33580-7 (PMC9588045; doi:10.1038/s41467-022-33580-7)
Supplement: Supplementary file 3 — Reporting Summary [file 41467_2022_33580_MOESM3_ESM.pdf]

nature portfolio

Corresponding author(s): Tong (Tina) Liu, PhD

Last updated by author(s): Aug 22, 2022

Reporting Summary

Nature Portfolio wishes to improve the reproducibility of the work that we publish. This form provides structure for consistency and transparency in reporting. For further information on Nature Portfolio policies, see our Editorial Policies and the Editorial Policy Checklist.

Statistics

For all statistical analyses, confirm that the following items are present in the figure legend, table legend, main text, or Methods section.

n/a	Confirmed	
☐	☒	The exact sample size (*n*) for each experimental group/condition, given as a discrete number and unit of measurement
☐	☒	A statement on whether measurements were taken from distinct samples or whether the same sample was measured repeatedly
☐	☒	The statistical test(s) used AND whether they are one- or two-sided<br>*Only common tests should be described solely by name; describe more complex techniques in the Methods section.*
☒	☐	A description of all covariates tested
☐	☒	A description of any assumptions or corrections, such as tests of normality and adjustment for multiple comparisons
☐	☒	A full description of the statistical parameters including central tendency (e.g. means) or other basic estimates (e.g. regression coefficient) AND variation (e.g. standard deviation) or associated estimates of uncertainty (e.g. confidence intervals)
☐	☒	For null hypothesis testing, the test statistic (e.g. *F*, *t*, *r*) with confidence intervals, effect sizes, degrees of freedom and *P* value noted<br>*Give P values as exact values whenever suitable.*
☐	☒	For Bayesian analysis, information on the choice of priors and Markov chain Monte Carlo settings
☒	☐	For hierarchical and complex designs, identification of the appropriate level for tests and full reporting of outcomes
☒	☐	Estimates of effect sizes (e.g. Cohen's *d*, Pearson's *r*), indicating how they were calculated

Our web collection on statistics for biologists contains articles on many of the points above.

Software and code

Policy information about availability of computer code

Data collection	MATLAB 2016b (MathWorks, MA) and MGL toolbox version 2.0 (http://gru.stanford.edu/doku.php/mgl/overview) were used to present the stimuli.
Data analysis	Publicly available software packages were used for preprocessing and analysis, including AFNI version 21.1.02 (https://afni.nimh.nih.gov/) for preprocessing of 3T BOLD data, mrTools version 4.7 (https://github.com/justingardner/mrTools) for preprocessing of 7T BOLD and 7T VASO data and analysis of all fMRI data, Freesurfer version 6.0 (http://surfer.nmr.mgh.harvard.edu/) and a probabilistic atlas version 0.10.1 (https://github.com/noahbenson/neuropythy/ for ROI-based analysis in Fig. 1 and retinotopic analysis in Fig. 4, LayNii toolbox version 1.0.0 (https://github.com/layerfMRI/LAYNII) for extracting cortical layers (LN_GROW_LAYERS program) in Fig. 3, and AFNI program RBA version 1.0.9 (https://afni.nimh.nih.gov/pub/dist/doc/program_help/RBA.html) and R package mgcv version 1.8-36 (https://cran.r-project.org/web/packages/mgcv/index.html) for Bayesian multilevel modeling in Figs. 1, 3 & 4. Customized code, source data, high-resolution figures, and computational simulation of different experimental designs are available on Zenodo (DOI: 10.5281/zenodo.7017856).

For manuscripts utilizing custom algorithms or software that are central to the research but not yet described in published literature, software must be made available to editors and reviewers. We strongly encourage code deposition in a community repository (e.g. GitHub). See the Nature Portfolio guidelines for submitting code & software for further information.

Data

Policy information about availability of data

All manuscripts must include a data availability statement. This statement should provide the following information, where applicable:

- Accession codes, unique identifiers, or web links for publicly available datasets
- A description of any restrictions on data availability
- For clinical datasets or third party data, please ensure that the statement adheres to our policy

The datasets generated during the current study are freely and publicly available to readers via figshare repository (doi: 10.6084/m9.figshare.14519127).

Human research participants

Policy information about studies involving human research participants and Sex and Gender in Research.

Reporting on sex and gender	The findings do not apply only to one gender/sex.<br>The non-pregnant female and male participants are defined by biological sex in our study.
Population characteristics	Data from 25 healthy right-handed volunteers (average age 25.9 years, 12 non-pregnant females, see Table 1) from the DC/MD/VA tri-state area were reported in this series of experiments (7T VASO, 7T BOLD, and 3T BOLD).
Recruitment	Subjects were recruited via advertisements in and around the NIH and local Bethesda, MD community. Due to the nature of the scanning environment at 7T, which features a narrower scanner bore and higher intensity of magnetic field than experiments at 3T, when possible, we gave preference to subjects who had participated in 7T studies in the past (or at least several 3T studies). This focus on experienced 7T participants for better data quality, a potential self-selection bias, likely resulted in cleaner data relative to a true community sample.
Ethics oversight	The protocol (93-M-0170, ClinicalTrials.gov identifier: NCT00001360) was approved by NIH Institutional Review Board. All participants granted informed consent.

Note that full information on the approval of the study protocol must also be provided in the manuscript.

Field-specific reporting

Please select the one below that is the best fit for your research. If you are not sure, read the appropriate sections before making your selection.

☒ Life sciences ☐ Behavioural & social sciences ☐ Ecological, evolutionary & environmental sciences

For a reference copy of the document with all sections, see nature.com/documents/nr-reporting-summary-flat.pdf

Life sciences study design

All studies must disclose on these points even when the disclosure is negative.

Sample size	The data reported in this study include 43 scan sessions from 25 participants (average age 25.9 years, 12 females, see Table 1), consisting of 14 scan sessions from 14 unique participants at 3T BOLD, 14 scan sessions from 14 unique participants at 7T BOLD, and 15 scan sessions from 10 unique participants at 7T VASO, among whom 5 were scanned twice to evaluate test-retest reliability of VASO. The sample size was chosen based on norms in the field. Our sample size is no smaller than those in many similar human fMRI studies, especially layer fMRI studies.
Data exclusions	A total of 53 2-hour scan sessions from 34 healthy right-handed volunteers (age 21-42 years, 16 females) from the DC/MD/VA tri-state area were collected in this series of experiments (7T VASO, 7T BOLD, and 3T BOLD). Based on conservative head motion parameter estimates across different magnetic strength or voxel size, seven 7T VASO scan sessions from six participants were excluded due to excessive head motion (>1 mm translation or >1° rotation within each run and/or >2 mm translation or >2° rotation across runs within a single scan session). Data from an additional 3 participants were further excluded because of technical errors, lack of scan time, or outlier behavioral performance (>3 SD below mean accuracy). Hence, the final dataset reported here includes a total of 43 scan sessions from 25 participants (average age 25.9 years, 12 females, see Table 1), consisting of 14 scan sessions from 14 unique participants at 3T BOLD, 14 scan sessions from 14 unique participants at 7T BOLD, and 15 scan sessions from 10 unique participants at 7T VASO, among whom 5 were scanned twice to evaluate test-retest reliability of VASO (see Supplementary Fig. 6).
Replication	11 out of 25 participants participated in more than one scan session across three experiments (3T BOLD, 7T BOLD, 7T VASO).<br><br>1. fMRI replication:<br>Eight healthy volunteers were scanned in both 3T BOLD and 7T BOLD experiments (S1, S3, S4, S15, S17, S18, and S20, see Table 1). Test-retest reliability of fMRI response to fearful - neutral faces (negative valence effect) across field strengths are available in Supplementary Figures 2-3.<br>Five healthy volunteers were invited back for a second scan in 7T VASO experiment (S4, S14, S15, S21, S22, see Table 1). Within-subject test-retest reliability of VASO results across scan sessions is available in Supplementary Figure 5.

2. Replication of behavioral task performance during the scan
Test-retest reliability of task performance showed that behavior was highly consistent across scan sessions on different days within participants (Supplementary Fig. 8).

Randomization: Randomization is not applicable since there were no groups in this study. This study adopts within-subject design.

Blinding: Blinding is not applicable since there were no groups in this study. This study adopts within-subject design.

Reporting for specific materials, systems and methods

We require information from authors about some types of materials, experimental systems and methods used in many studies. Here, indicate whether each material, system or method listed is relevant to your study. If you are not sure if a list item applies to your research, read the appropriate section before selecting a response.

Materials & experimental systems

n/a	Involved in the study	
☒	☐	Antibodies
☒	☐	Eukaryotic cell lines
☒	☐	Palaeontology and archaeology
☒	☐	Animals and other organisms
☒	☐	Clinical data
☒	☐	Dual use research of concern

Methods

n/a	Involved in the study	
☒	☐	ChIP-seq
☒	☐	Flow cytometry
☐	☒	MRI-based neuroimaging

Magnetic resonance imaging

Experimental design

Design type: Task, block design

Design specifications: In both 3T BOLD and 7T BOLD experiments, each run consisted of three repeats of each facial expression condition (fearful, neutral, happy) and ten repeats of the fixation block. Within each facial expression block, each face was presented for 900 ms with a 100 ms interstimulus interval (ISI) while a green fixation cross remained at the center of the screen at all times. Each fixation block lasted 10 s and each face block lasted 20 s in the 3T BOLD experiment. Hence, each run lasted 4 min 40 s in total. Similarly, each fixation block lasted 9 s and each face block lasted 18 s in the 7T BOLD experiment; thus each run lasted 4 min 12 s in total.

In the 7T VASO experiment, each run consisted of six repeats of each facial expression condition (fearful, neutral, happy) and 19 repeats of the fixation blocks. There were 16 faces presented in each face block. Each face was shown for 1100 ms with a 106.25 ms ISI. Thus, each face block lasted 19.3 s and each fixation block lasted for 9.65 s, and each run lasted 8 min 53 s.

Behavioral performance measures: Participants performed a gender judgment task (press "1" for female, "2" for male) for each face stimulus, unrelated to facial expressions. Feedback on task performance (percent correct) and real-time head motion estimates were given to the participant shortly after each run; no feedback was given during scanning.

There was no significant difference in gender judgment accuracy across the three fMRI experiments (3T BOLD: 92.07 ±3.90%, 7T BOLD: 91.66±3.31%, 7T VASO: 92.35±4.71%, one-way ANOVA: $F(2,35) = 0.094$, $P = 0.910$, Supplementary Fig. 7a). To examine potential within-subject performance differences across facial expressions, we collapsed performance within each of those participants who participated in multiple scan sessions (see Table 1). There was a significant main effect of facial expression on gender judgment performance, consistent across accuracy, reaction time (RT, correct trials only), and inverse efficiency score (IES) measures (one-way repeated measures ANOVA: all P values < 0.001). Specifically, performance when fearful faces were presented (accuracy: 89.75±4.18%, RT: 641±46ms, IES: 715±56ms) was significantly worse than performance when neutral (accuracy: 92.83±3.82%, RT: 637±42ms, IES: 687±53ms) or happy faces (accuracy: 94.53±3.03%, RT: 631±43ms, IES: 668±47ms) were presented (Supplementary Fig. 7b-d). Importantly, test-retest reliability of task performance showed that behavior was highly consistent across scan sessions on different days within participants (Supplementary Fig. 8).

Acquisition

Imaging type(s): BOLD, VASO

Field strength: 3T, 7T

Sequence & imaging parameters: BOLD scan parameters:
3T BOLD fMRI data were acquired using multi-echo gradient-echo echo planar (EPI) sequence (TR = 2000 ms, TE1 = 12.5 ms, TE2 = 27.6 ms, TE3 = 42.7 ms, voxel size = 3.2 x 3.2 x 3.5 mm, flip angle = 75°, echo spacing = 0.4 ms, grid size = 64 x

64 voxels, 30 slices).
7T BOLD fMRI data were acquired using a gradient-echo EPI sequence (TR = 1500 ms, TE = 23 ms, voxel size = 1.2 x 1.2 x 1.2mm, flip angle = 55°, grid size = 160x160 voxels, 42 slices).

VASO scan parameters:
7T VASO data were acquired using an inversion recovery prepared 3D-EPI sequence, which was optimized for layer-specific fMRI in human visual cortex. Parameters of inversion recovery preparation were as follows: The adiabatic VASO inversion pulse is based on the TR-FOCI pulse, with a duration of 10 ms and a bandwidth of 6.3 kHz. The inversion-efficiency was adjusted by the implementation of a phase skip of 30 deg to minimize the risk of inflow of fresh non-inverted blood into the imaging region during the blood nulling time. 7T VASO data were acquired using a 3D-EPI readout with the following parameters: 0.82 x 0.82 x 0.82 mm, FOV read=133mm, 26 slices, whole k-space plane acquired after each shot, FOV in first phase encoding direction = 133.3% of FOV in readout direction, TE=24ms, GRAPPA 3, partial Fourier of 6/8. To account for the T1-decay during the 3D-EPI readout and potential related blurring along the segment direction, a variable flip angle (FA) was applied across segments, which started from 22°, and then exponentially increased until reaching a desired flip angle of 90°.

The acquired time series consisted of interleaved BOLD and VASO images, with TR(BOLD)=2737ms and TR(VASO)=2088 ms, resulting in effective TR(VASO+BOLD)=4825ms. Details of the 7T sequence and scan parameters can be found: https://github.com/tinaliutong/sequence.

Imaging slice position and angle were adjusted individually for each 7T VASO participant so that the slice prescription was parallel to each participant's calcarine sulcus (visualized on the sagittal plane prior to the scan, see Supplementary Fig. 6a). We also ran the retinotopic atlas analysis based on each participant's T1-weighted MPRAGE MRI, acquired in a separate session prior to the main experimental scan session. This was used to guide slice prescription, aiming to maximally cover V1 in each participant. After slice prescription, a 3rd order B0-shimming was done with four iterations. The shim volume was parallel to the slice prescription.

Image reconstruction was done in the vendor-provided platform (Siemens software identifier: IcePAT WIP 571) and was optimized with the following set-up to minimize image blurring and increase tSNR at high resolution. GRAPPA kernel fitting was done on FLASH autocalibration data with a 3x4 kernel, 48 reference lines and regularization parameter χ= 0.1. Partial Fourier reconstruction was done with the projection onto convex sets (POCS) algorithm with eight iterations. Data of each coil channel were combined with the sum-of-squares.

Structural MRI
Within the same 3T scan session, anatomical images were acquired in each individual for co-registration purpose using a 3D Magnetization-Prepared Rapid Acquisition Gradient Echo (MPRAGE) sequence with 1 mm isotropic voxels, 176 sagittal slices, acquisition matrix = 256 x 256, TI/TE/TR = 900/1.97/2300 ms, flip angle = 9°, GRAPPA = 2, scan time = 5 min 21s. The 3T anatomy was also used for co-registration of all 3T BOLD participants and 8 of 14 7T BOLD participants (who participated in both 3T BOLD and 7T BOLD scans). In other 7T participants, a 0.7 mm isotropic resolution T1-maps were collected covering the entire brain using an MP2RAGE sequence with TI1/TI2/TR/TE = 800/2700/6000/3.02 ms, FA1/FA2 = 4°/5°, 224 sagittal slices, matrix size = 320 × 320, scan time = 10 min 8 s. Before the VASO scan, we made sure all participants had prior MPRAGE data available, which was used to estimate the slice angle of the VASO scan.

Area of acquisition

3T BOLD: Whole-brain coverage
7T BOLD: Up to 2/3 whole-brain coverage with full coverage of the temporal and occipital lobes and partial coverage of the parietal and frontal lobes.
7T VASO: Given that the high-resolution fMRI pulse sequences used in this study currently do not permit a whole-brain field of view, we guided slice prescription to maximally cover V1 in this experiment. Imaging slice position and angle were adjusted individually for each 7T VASO participant so that the slice prescription was parallel to each participant's calcarine sulcus (visualized on the sagittal plane prior to the scan, see Supplementary Fig. 6a). We also ran the retinotopic atlas analysis based on each participant's T1-weighted MPRAGE MRI, acquired in a separate session prior to the main experimental scan session.

Diffusion MRI ☐ Used ☒ Not used

Preprocessing

Preprocessing software

All preprocessing steps were implemented in MATLAB 2016b using a combination of mrTools78 and AFNI software package. Standard preprocessing of the 3T multi-echo gradient echo EPI data utilized the AFNI software program afni_proc.py. Data from the first 4 TRs were removed to allow for T1 equilibration and to allow the hemodynamic response to reach steady state. Advanced automatic denoising was achieved using multi-echo EPI imaging and analysis with spatial independent component analysis (ICA), or ME-ICA. Preprocessing of 7T BOLD data included head movement compensation within and across runs, linearly detrended, and high-pass filtered (cutoff: 0.01 Hz) to remove low-frequency noise and drift. For 7T VASO data, all time frames were first split into blood-nulled and blood-not-nulled (BOLD) groups. Motion correction was performed separately for each group. The time frames from each group were upsampled in time via cubic interpolation and the first and last two upsampled time frames in each group were removed from each run. Next, CBV-weighted VASO signals were calculated as blood-nulled divided by blood-not-nulled (BOLD) at each time frame to remove BOLD contamination.

Normalization

No normalization was applied. All data analysis was conducted in native space.

7T VASO experiment: To ensure the most accurate definition of cortical depths, we used the functional 7T VASO data directly to generate an anatomical reference, termed VASO anatomy. It was computed through dividing the inverse signal variability across blood-nulled and blood-not-nulled images by mean signals. This measure is also called T1-EPI, which provides a good contrast between white matter (WM), gray matter (GM) and cerebral spinal fluid (CSF; see Fig. 3b) in native EPI space.

	3T and 7T experiment: For visualization purposes, we applied the freesurfer average cortical surface template to visualize the valence effect on the inflated cortical surface at the group level (Figure 1b, Supplementary Figures 1 and 4)
Normalization template	freesurfer average cortical surface template was used for visualizing group effects (Figure 1b, Supplementary Figures 1 and 4)
Noise and artifact removal	3T BOLD: Data from the first 4 TRs were removed to allow for T1 equilibration and to allow the hemodynamic response to reach steady state. Advanced automatic denoising was achieved using multi-echo EPI imaging and analysis with spatial independent component analysis (ICA), or ME-ICA.<br><br>7T BOLD: Data were head movement compensated within and across runs, linearly detrended, and high-pass filtered (cutoff: 0.01 Hz) to remove low-frequency noise and drift.<br><br>7T VASO: All time frames were first split into blood-nulled and blood-not-nulled (BOLD) groups. Motion correction was performed separately for each group. The time frames from each group were upsampled in time via cubic interpolation and the first and last two upsampled time frames in each group were removed from each run. Next, CBV-weighted VASO signals were calculated as blood-nulled divided by blood-not-nulled (BOLD) at each time frame to remove BOLD contamination.
Volume censoring	No volume censoring was conducted.

Statistical modeling & inference

Model type and settings	Standard general linear model (GLM) analyses were performed. The regressor for each condition of interest (faces, objects, and scrambled objects in the face localizer task, or fearful, neutral, happy in the gender judgement task) was convolved with a canonical hemodynamic response function.<br><br>A region-based analysis was performed through Bayesian Multilevel (BML) modeling through the AFNI program RBA (https://afni.nimh.nih.gov/pub/dist/doc/program_help/RBA.html). The BML modeling results are presented with each region's posterior distribution (Fig. 1c, Supplementary Fig. 4b). Each contrast between two conditions C1 and C2 was expressed as a dimensionless modulation index (C1-C2)/(|C1|+|C2|), whose posterior distribution was represented through the posterior samples drawn from the Markov chain Monte Carlo simulations of the BML model. The strength of statistical evidence is shown through P+, the posterior probability of each region's effect being positive conditioning on the adopted BML model and the current data. See the BML model performance in Supplementary Fig. 4c. See Methods for equations and details.
Effect(s) tested	The correlation coefficients between each pair of ROIs, for fearful and neutral conditions, were computed based on the residual time series (measured response time series - predicted response time series estimated using deconvolution) (Fig. 2a) and their difference in correlation (fearful - neutral) was entered into Wilcoxon signed-rank test (Fig. 2b). The beta weights (in units of percent signal change) and t statistics for the fearful, happy, and neutral conditions were entered into Bayesian Multilevel (BML) modeling (Figs. 3-4; Supplementary Figs. 5-6).

Specify type of analysis: ☐ Whole brain ☒ ROI-based ☐ Both

Anatomical location(s)	3T BOLD and 7T BOLD: We segmented the visual cortex into 13 regions of interest (ROIs, labeled in Fig. 1b-c) using a probabilistic retinotopic atlas (Wang et al., 2005, doi: 10.1093/cercor/bhu277). First, 25 visual areas per hemisphere were defined in these 23 scan sessions (from 15 unique participants). Next, visual areas with the same area label were combined across hemispheres (with IPS1-5, LO1-LO2, PHC1-PHC2, TO1-TO2, V1d-V1v, V2d-V2v, V3A-V3B, V3d-V3v, VO1-VO2 combined) and were further thresholded by R2 value in the independent face localizer (R2>0.1 at both 3T BOLD and 7T BOLD). We functionally defined the amygdala and the FFA using an independent localizer (see Methods).<br><br>3T BOLD, 7T BOLD, and 7T VASO: We performed a retinotopic analysis (Fig. 4a, Supplementary Fig. 6a) using a probabilistic atlas (Benson et al., 2014, https://doi.org/10.1371/journal.pcbi.1003538) to segment V1 based on visual eccentricity (deg). The eccentricity map was visualized on a flat patch of early visual cortex and a portion of central V1 corresponding to the size and position of the face stimuli was highlighted by the yellow contour (Fig. 4a-b).

Statistic type for inference<br>(See [Eklund et al. 2016](#))	No voxel-wise inference was drawn in this study except defining functional ROIs using the independent face localizer. The amygdala and the FFA were functionally defined from the independent face localizer using a conjunction between t map of faces-objects (whole brain FDR<0.05) and R2 map (R2>0.1 for 11 scan sessions at 3T BOLD or R2>0.05 for 12 scan session at 7T BOLD). fMRI response from the 13 anatomical ROIs in the visual cortex and functionally defined amygdala and FFA (in Figure 1 and Supplementary Figure 4) were thresholded by R2 value in the independent face localizer (R2>0.1 at both 3T BOLD and 7T BOLD).
Correction	Multiple comparison was performed for number of ROIs in Figure 1, for number of cortical depths in Figure 3 and for number of eccentricity bins in Figure 4.

Models & analysis

n/a	Involved in the study
☐	☒ Functional and/or effective connectivity
☒	☐ Graph analysis
☒	☐ Multivariate modeling or predictive analysis

Functional and/or effective connectivity	Pearson correlation coefficients between each pair of ROIs (defined above) were computed from the residual time series in each ROI corresponding to each facial expressions condition (Fig. 2a). The differences (fearful – neutral) and (happy - neutral) in correlations were also computed (Fig. 2b-c). For participants who were scanned in multiple sessions, correlation coefficients were averaged between sessions.
