# Supplementary material for: Layer-specific, retinotopically-diffuse modulation in human visual cortex in response to viewing emotionally expressive faces
Source: Nat Commun. 2022 Oct 22;13:6302. doi: 10.1038/s41467-022-33580-7 (PMC9588045; doi:10.1038/s41467-022-33580-7)
Supplement: Supplementary file 2 — Peer Review File [file 41467_2022_33580_MOESM2_ESM.pdf]

nature portfolio

Peer Review File

Layer-specific, retinotopically-diffuse modulation in human visual cortex in response to viewing emotionally expressive faces

Open Access This file is licensed under a Creative Commons Attribution 4.0 International License, which permits use, sharing, adaptation, distribution and reproduction in any medium or format, as long as you give appropriate credit to the original author(s) and the source, provide a link to the Creative Commons license, and indicate if changes were made. In the cases where the authors are anonymous, such as is the case for the reports of anonymous peer reviewers, author attribution should be to 'Anonymous Referee' followed by a clear attribution to the source work. The images or other third party material in this file are included in the article's Creative Commons license, unless indicated otherwise in a credit line to the material. If material is not included in the article's Creative Commons license and your intended use is not permitted by statutory regulation or exceeds the permitted use, you will need to obtain permission directly from the copyright holder. To view a copy of this license, visit http://creativecommons.org/licenses/by/4.0/.

Reviewers' comments:

Reviewer #1 (Remarks to the Author):

This study investigates the cortical depth specificity of emotional faces (fearful, neutral, and happy) in humans using VASO contrast at 7T. In addition, using the same stimuli, at 3T and 7T, BOLD signal activation across many areas (visual cortex, amygdala) were measured. It was found that the VASO contrast differences related to facial valence was largest in superficial depths in early visual cortex, suggesting feedback processing, and BOLD signal differences were larger in spatial extent than the retinotopic correspondence of the presented faces, suggesting a general diffuse modulation of neuronal activity. These two findings were interpreted as being caused by feedback from amygdala projections into the visual cortex.

In general, it is a valuable goal to investigate feedback processing in the early visual cortex, which is currently understudied. In particular, cortical depth specific MRI, a relatively new tool for human cognitive neuroscience, promises to provide insights into this topic. However, in the current study the evidence provided does not sufficiently support the claim of amygdala-caused feedback processing of face valence in the early visual cortex. In detail, more robust statistical analyses have to be performed and additional results shown:

Major points:
1. The effect sizes shown in Fig. 2g and 3c, on which the main conclusion rely upon, are quite small. It is claimed that these are statistically significant. However, the authors test significance on the mean values within subjects, which are assumed to not have any error. In other words, the values shown in these Figures have errors themselves and any statistical test has to use standard error propagation laws. Given, for example, the high spatial variability shown in Fig 3b, it is reasonable to expect that the mean values for each subject in Figure 3c have large errors, which the statistical tests have to take into account. Please recalculate the statistical significance both for cortical depth and regional analyses.

2. Related to this point: Fig 3c and Suppl Fig 5, there is large inter-subject distribution of data, which is not unexpected but leads to the danger that the main results are outlier determined, e.g. the subject shown in yellow in Fig 3c or s14 in Suppl Fig 5 (both without error bars!) may bias the results. In almost all other subjects the main effects are not clearly visible. The authors claim that their statistical test take into account potential outliers but this is not obvious to the reviewer. Thus, the sample size may be too small to reduce outlier sensitivity. And: A permutation test including intra-subject variability may be more suitable for this purpose.

3. There is no direct evidence provided of amygdala feedback but inferred from the observation from the visual cortex alone, which leads to a highly speculative Discussion section. However, the data acquired may allow providing additional and more direct evidence. For example, from the whole brain 3T and 7T BOLD data, a trial-to-trial analysis can be performed to test whether amygdala activity and the claimed diffuse-feedback activity do actually covary, which they have to do according to this hypothesis. Please reanalyze the data with this perspective and present the results in the main manuscript.

4. To obtain cortical depth profile, the data is upsampled by 4 and then 21 cortical depths calculated over the ROIs. However, looking at Fig 2c and 2e, the number of voxels included in the VASO analysis seems to be low, questioning whether it is feasible to determine cortical depths in sufficient detail. Upsampling data to a higher resolution does not increase the information content of data. Please provide the number of original voxels in each subject included in the cortical depth analysis in a Table and discuss the potential impact of these numbers on the results.

5. Together with the VASO data, the BOLD signal was acquired with high spatial resolution. Please show the cortical depth profile for the same contrasts also using the BOLD signal and discuss commonalities and discrepancies. It is also written that: “By measuring CBV responses across cortical

layers (Fig. 2c-e), our approach enabled layer-specific measurements of V1 activity that are inaccessible with BOLD fMRI." This is a strange statement as most cortical depth fMRI studies are done using the BOLD contrast. Please explain why the authors think that BOLD fMRI is not useful for layer-specific studies.

6. The authors demonstrate that gender judgment task and face valence are correlated but dismiss the possibility that the gender task may (partially) explain the results: "An RSA on pixel-level discriminability between female and male faces in each expression group revealed a significant effect of expression on gender discriminability (one-way ANOVA: all F values > 148.03, all P values < 0.001 across Euclidian distance, correlation distance and cosine distance)." And: "… gender judgement task on the face stimuli, unrelated to facial expression". Please clarify what "unrelated" means in this context and provide statistical maps and results related to gender judgment (both for cortical depth and regional analysis).

7. The experimental design is suboptimal. The face blocks are twice as long in duration compared to the fixation blocks. Usually, the rest or control duration in fMRI studies is longer than the stimulus duration to allow the hemodynamic signals return to baseline, due to adaptation, post-stimulus inhibition and undershoot etc. Thus, the short fixation duration may influence the value of the baseline signal, which itself may have cortical depth specific effects. Ideally new data has to be acquired to rule out such possibility. Alternatively, the authors should discuss this issue in the Discussion section.

Minor:
1. Fig. 2d. What is "Response phase"? Please clarify.

Reviewer #2 (Remarks to the Author):

The authors investigated the valence effect (VE) in human primary visual cortex using VASO and BOLD fMRI. They derived a measure of VE at a cortical-depth dependent level as well at a retinotopic level.
Results indicate a cortical-depth dependent specificity of VE as well as a diffuse retinotopic effect, suggesting that valence information is processed in the amygdala and fed back to early visual cortex.

The main research question is of general interest and revolves around the investigation of feedback mechanisms affecting early visual cortex. From a methodological perspective the investigation is solid, and the data is convincing.

I believe several points should be addressed before granting publication regarding the implications of the experimental design adopted and the interpretation of the results.

Regarding the specificity of the amygdala/V1 pathway:
Based on the specific pattern of results observed, the authors conclude that these are compatible with the known anatomical connectivity between the amygdala and V1.
I agree with the authors that this might be the most likely pathway behind the observed results. However, the interpretation would be corroborated by also showing more specific link between the activity in the amygdala and V1 in the current data. For example, looking at amygdala-V1 correlations in the current dataset (low-resolution) compared to other areas, as V2-V1 or V3-V1 correlation. This type of question could be addressed at the functional connectivity level or as a correlation of the valence effect measured in the two areas.

Considering my comment above, I would suggest rephrasing or toning down this point in the discussion (pg. 13)

'Here, we use layer-specific fMRI to isolate a circuit arising from a subcortical area, the amygdala, that

plays a powerful role in shaping responses at the earliest stage of visual cortical processing.'

Alternative hypotheses put forward in the introduction and the discussion:
In the introduction two alternative hypotheses are clearly stated, leading to specific pattern of expected results.
In the discussion the pattern of results is interpreted with respect to three alternatives, one of which is referred to also in the introduction (the second possibility).
This difference between the number of alterative hypotheses in the introduction/discussion could create some confusion and should potentially be homogenised.

Positive valence:
For completeness of the analysis, I would suggest also reporting the results from the positive valence – neutral controls along depth. While these results are partially described in supplementary figure 4, I would dedicate a section about positive valence effect along cortical depth.

Eye movements:
Given the negative valence index shows such diverse distribution patterns across so many areas, is there a possibility there are multiple mechanisms at play? For example: eye movements were not monitored online (even if the task was just to fixate), could some of the activity (e.g. FEF) due to different eye movement patterns when presented with neutral faces/fearful faces?

fMRI contrast:
While it is clear that figure 2 shows VASO and 7T BOLD results, the same is not true for figure 1 and figure 3. Please report in the manuscript or the figure caption the field strength at which the data was collected (7T or 3T).

Gender judgement:
In page 4 the authors define the gender judgement task as demanding. The average accuracy across the three experiments range between 91 and 92%. I think this does not count as a demanding task, as it is almost at ceiling level. I would remove the term 'demanding'.

Supp fig. 7, legend:
I think from the legend (in gray) it is very difficult to guess which colour is supposed to correspond to which session, I would suggest reformatting this figure / legend with a more intuitive mapping.

Reviewer #3 (Remarks to the Author):

This study is well described, and uses novel acquisition and painstaking analysis approaches to address an interesting research question. I am unable to assess the reliability of the VASO technique to identify layer-specific activity, but the results are credible, despite the small sample of 10 subjects.

The primary concern I have with this paper is the unqualified premise that V1 is enhanced by emotional cues. The evidence for this is sparse, while the evidence against is at least as strong, if not stronger. Only the Pessoa paper lists V1 as sensitive to emotional quality. In the Lang '98 and Bradley '03 studies cited, there is no specific evidence for emotion effects in V1. These studies used natural scenes and analyses collapsed across entire coronal slices of the brain. Other similar scene studies that employed perceptually balanced emotional and neutral stimuli (Sabatinelli et al., 2009, J Nsci) explicitly test emotion effects and report no difference in BOLD signal in calcarine fissure. This is consistent with fMRI meta analyses of emotional face processing, which have not identified primary visual cortex. The Amaral research suggests strong connectivity from basal amygdala to TE, but weak connectivity with V1. The Yamamori & Rockland paper offers some evidence for thalamocortical connectivity with V1, but only suggestive evidence for amygdala-V1 connectivity. Thus the paper might include some dicsussion of this possibility in the introduction.

Other concerns.

1. How might the rapidity of ventral visual cortical activity affect V1 activity relative to the 2+ second sampling rate of fMRI? Could information flow through thalamocortical pathways more rapidly than be accounted for with hemodynamic measures?

2. Did subjects feel fear during the experiment?

3. How was gaze controlled or recorded in the MR scanner? Could saccades explain the nonretinotopic effects?

4. Could this be a face-specific effect? As the authors state, the processing of emotional expressions is a multifaceted socio-communicative behavior, distinct from emotion in general.

December 22, 2021

We thank the reviewers for their insightful comments and suggestions regarding our manuscript "**Layer-specific, retinotopically-diffuse modulation in human visual cortex by emotional faces.**" We have incorporated all of their suggestions in the revised manuscript, including new analyses, substantial revisions of the text, and the clarification of a range of issues raised in the reviews. Below, we start by summarizing three of the biggest changes to this revision, which address broad issues raised by the reviewers. We then provide a point-by-point response to each of their comments. For ease of reference, reviewers' comments have been numbered consecutively and are indicated in blue. We also provide pointers to specific page numbers and figures in the revised manuscript, and excerpts from the revised text where appropriate. We believe that we have addressed all of the reviewers concerns and in so doing have greatly strengthened this study.

Summary of revision:

1. Relevance of amygdala-V1 connectivity. All three reviewers (and the editor) questioned whether V1 was the most logical place to study the impact of feedback from the amygdala. Reviewers 1 & 2 felt that a functional connectivity analysis would help support a role for amygdala-V1 feedback in facial valence processing, while Reviewer 3 asked about the 'unqualified premise that V1 is enhanced by emotional cues'. To address these concerns, we have added a major new functional connectivity analysis (**new Figure 2**) demonstrating that viewing fearful faces is associated with enhanced correlation in the residual time series between amygdala and V1 (both central V1, where the stimulus was presented, and peripheral V1, beyond the spatial extend of the face stimuli). We did not, however, observe inter-area correlation enhancement between V1 and other visual areas, suggesting that the increased connectivity was specific to the amygdala, and did not arise from intracortical feedback from other visual areas.

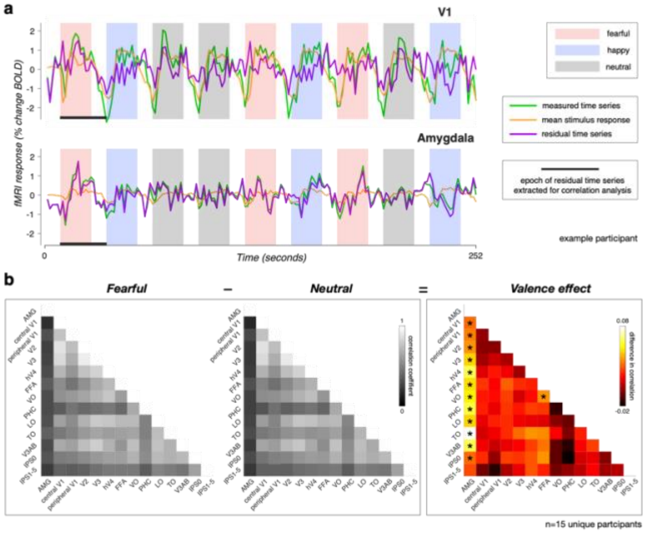

New Figure 2: Inter-area correlation reveals enhanced interactions with amygdala when viewing fearful faces. a, fMRI time series from V1 (top) and the amygdala (bottom) from a single run from an example participant in the 7T BOLD experiment, consisting of three 18 s blocks of trials of each facial expression (pink: fearful; blue: happy; gray: neutral) with interleaved blocks of fixation of 9 s. Three time series are plotted: green, measured time series; orange, mean stimulus-evoked response (estimated using deconvolution); purple, residual time series after removing the mean stimulus-evoked response. Horizontal black bars indicate the epoch of residual time series that was extracted for correlation analysis. **b**, Correlation coefficients for fearful face condition (left) and neutral face condition (middle), and the differences in correlation (fearful − neutral), indicating the valence effect (right). Each square in grayscale indicates the correlation between residual time series for a pair of ROIs (left-middle). Each square in color indicates the difference in correlation between fearful and neutral conditions for a pair of ROIs (right). Asterisks represent ROI pairs showing a statistically significant difference in correlation (*$P < 0.05$, Wilcoxon signed-rank test). Number of unique participants scanned at 3T BOLD and 7T BOLD who were also scanned in face localizer experiment: n=15 (see Table 1).

2. Statistical procedures. Reviewer 1 had a number of helpful suggestions regarding our statistical modeling procedures, and especially the propagation of individual subject variance in the group-level statistics. In the revision, we have, with the assistance of co-author Gang Chen, implemented a hierarchical Bayesian modeling framework[1–3] that addresses these concerns. We have used this new model extensively in the revision (see **new Figure 3f-g, new Figure 4c, and new Supplementary Figures 4-5)**.

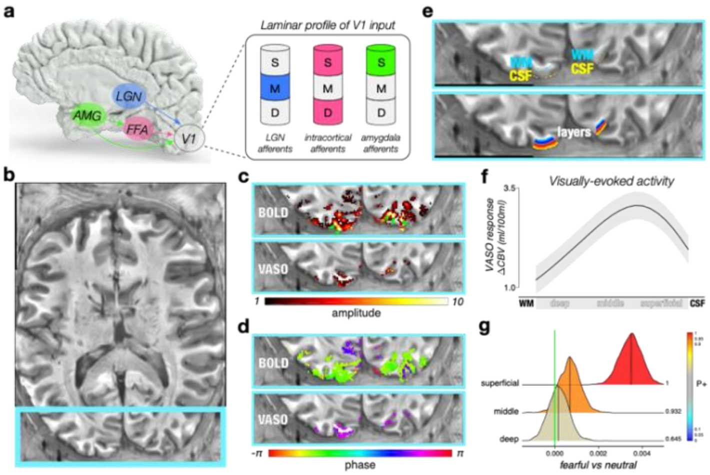

New Figure 3. **Facial valence modulation specific to superficial layers of V1. a**, Three input pathways to V1 have distinct laminar profiles: LGN afferents terminate in the middle layer[49,50], cortico-cortical afferents, such as from FFA, terminate in the superficial and deep layers[4], and amygdala afferents terminate exclusively in the superficial layer[4,51]. AMG: amygdala, FFA: fusiform face area, LGN: lateral geniculate nucleus, D: deep layer, M: middle layer, S: superficial layer. **b**, Axial slice of a T1-weighted anatomical image generated from VASO timeseries[14]. Light blue line corresponds to field of view shown in panels c-e. **c**, Response amplitude to face stimuli measured with BOLD (top) and VASO (bottom). VASO measurements are lower in amplitude but are more closely colocalized with cortical gray matter. Green arrows in the BOLD image (top) indicate high-amplitude responses in veins. **d**, Phase (timing) of best-fitting sinusoid. BOLD (top) and VASO (bottom) are known to have opposite signed responses[14], as indicated by the 180 deg shift in response phase. **e**, The central V1 ROI was defined in each participant based on retinotopic analysis[52] and further constrained by demarcating the white matter (WM; cyan) and CSF (yellow) boundaries (top). Between these WM and CSF boundaries, 21 cortical depths were generated with LAYNII software[53] (bottom). **f**, Percent change in VASO (in units of ml per 100 ml CBV) to all faces (pooled across fearful, neutral and happy expressions) as a function of relative cortical depth between WM (left) and CSF (right). The black line shows the fitted average cross-layer profile among the three conditions while the shaded band indicate the uncertainty range of one standard error. **g**, Posterior distribution of fearful – neutral VASO responses (in units of ml per 100 ml CBV) as a function of cortical depth. Hue indicates the strength of statistical evidence according to the Bayesian Multilevel (BML) model[3] (see Methods), shown through *P+* (value at right side of each posterior distribution), the posterior probability of the valence effect at each cortical depth's being positive conditioning on the adopted BML model and the current data. The vertical green line indicates zero effect. Cortical depths with strong evidence of the valence effect can be identified as the extent of the green line being farther into the tail of the posterior distribution. **f-g**, Number of unique participants scanned at 7T VASO: n=10 (15 scan sessions, see Table 1).

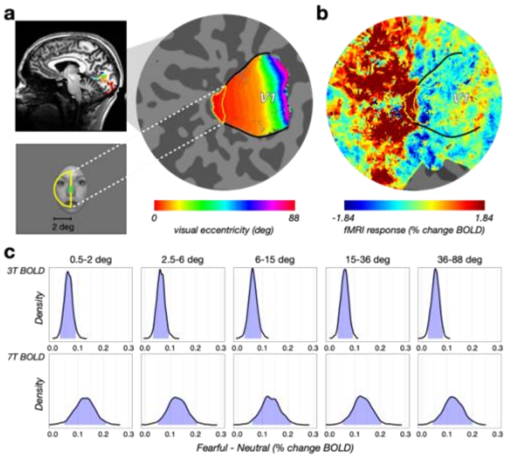

New Figure 4. **Facial valence modulation as a function of visual eccentricity. a**, Face stimuli subsumed a 4 deg x 6 deg ellipse centered at the fovea and were expected to evoke responses in a retinotopically-identified region of V1, shown in a mid-sagittal slice (top left) and on a computationally flattened patch of early visual cortex (right). Hue indicates visual eccentricity. Yellow contour on the flat map indicates retinotopic location of face stimulus (bottom left). **b**, Visually-evoked BOLD response to all faces (same participant as in panel a). Black curves indicate V1/V2 boundary. Spatial pattern of visual response exhibits a strong positive response at retinotopic location of the stimulus (red voxels), a surrounding negative penumbra at mid-eccentricities (dark blue voxels), and a return to baseline at far eccentricities (cyan, green, and yellow voxels). **c**, Valence modulation evident at all visual eccentricities. The statistical evidence for the elevated activity in response to fearful relative to neutral faces was substantial at all visual eccentricities. Under the posterior distribution of each eccentricity bin, the blue shadow indicates the 95% uncertainty intervals of the valence effect (fearful – neutral) with 5 eccentricity bins, separately for 3T BOLD (top) and 7T BOLD (bottom) scans. Number of participants scanned at 3T BOLD: *n*=14; number of participants scanned at 7T BOLD: *n*=14 (see Table 1).

3. Contrast mechanism for layer fMRI. Reviewer 1 questioned the relative advantages of using VASO over BOLD fMRI for making inferences about the laminar profile of cortical activity. This issue can best be viewed as a bias/variance tradeoff. BOLD fMRI has high sensitivity but is known to be heavily biased by draining veins toward superficial layers, which obfuscates measurement of true layer-specific activity differences. VASO fMRI is much noisier but provide a more unbiased measure of activity across cortical depths. VASO is a relatively new technique, and we feel that our use of VASO is a strength. We have greatly expanded our discussion of this important and rapidly evolving set of issues in the revision.

Reviewer 1:

This study investigates the cortical depth specificity of emotional faces (fearful, neutral, and happy) in humans using VASO contrast at 7T. In addition, using the same stimuli, at 3T and 7T, BOLD signal activation across many areas (visual cortex, amygdala) were measured. It was found that the VASO contrast differences related to facial valence was largest in superficial depths in early visual cortex, suggesting feedback processing, and BOLD signal differences were larger in spatial extent than the retinotopic correspondence of the presented faces, suggesting a general diffuse modulation of neuronal activity. These two findings were interpreted as being caused by feedback from amygdala projections into the visual cortex.

In general, it is a valuable goal to investigate feedback processing in the early visual cortex, which is currently understudied. In particular, cortical depth specific MRI, a relatively new tool for human cognitive neuroscience, promises to provide insights into this topic. However, in the current study the evidence provided does not sufficiently support the claim of amygdala-caused feedback

processing of face valence in the early visual cortex. In detail, more robust statistical analyses have to be performed and additional results shown:

Response: We thank this reviewer for the positive evaluation of our manuscript and for the helpful comments and suggestions for how to improve it.

Major points:

R1.1 The effect sizes shown in Fig. 2g and 3c, on which the main conclusion rely upon, are quite small. It is claimed that these are statistically significant. However, the authors test significance on the mean values within subjects, which are assumed to not have any error. In other words, the values shown in these Figures have errors themselves and any statistical test has to use standard error propagation laws. Given, for example, the high spatial variability shown in Fig 3b, it is reasonable to expect that the mean values for each subject in Figure 3c have large errors, which the statistical tests have to take into account. Please recalculate the statistical significance both for cortical depth and regional analyses.

Response: The reviewer is raising an important statistical point here, with which we wholly agree. Most fMRI studies use random-effects analyses in which each participant contributes a single point estimate and the group-level models only incorporate variance across participants. This is the practice that we followed in our initial submission. But this approach throws out within-subject variance, which may be problematic when the point estimates are themselves noisy (as in layer-specific VASO measurements). To address this concern, following the reviewer's suggestion, we have reperformed all of the statistical analyses shown in **new Figure 3** and **new Figure 4** using a hierarchical Bayesian model[1,3] that takes into account the contribution of subject-level variability. All of our conclusions remain valid under this new modeling framework.

R1.2 Related to this point: Fig 3c and Suppl Fig 5, there is large inter-subject distribution of data, which is not unexpected but leads to the danger that the main results are outlier determined, e.g. the subject shown in yellow in Fig 3c or s14 in Suppl Fig 5 (both without error bars!) may bias the results. In almost all other subjects the main effects are not clearly visible. The authors claim that their statistical test take into account potential outliers but this is not obvious to the reviewer. Thus, the sample size may be too small to reduce outlier sensitivity. And: A permutation test including intra-subject variability may be more suitable for this purpose.

Response: The reviewer again raises a good point about the statistical modeling, and specifically whether the effects are driven by outlier subjects. The Bayesian Multilevel (BML) modeling[1,3] that we use in the revision addresses issues arising from potential outliers and large inter-subject distributions. Specifically, two features of the modeling framework are helpful in dealing with outliers: 1) a student *t*-distribution accommodates potential outliers and skewness in the data; 2) the incorporation of standard errors in the model also mitigates concerns about individual outlier datapoints.

As a complementary approach, in order to be absolutely certain that the results were not driven by a single outlier, we reran the original statistical analysis from the first submission both with and without the outlier subject that the reviewer noted. Importantly, we observed the same pattern of results.

Including the outlier subject:

0.5-2 deg: $t(19) = 3.593$, $p = 0.002$
2.5-6 deg: $t(19) = 3.492$, $p = 0.002$

6-15 deg: t(19) = 3.658, p = 0.002
15-33 deg: t(19) = 3.917, p < 0.001
33-88 deg: t(19) = 3.073, p = 0.006

Excluding the outlier subject:
0.5-2 deg: t(18) = 3.401, p = 0.003
2.5-6 deg: t(18) = 3.582, p = 0.002
6-15 deg: t(18) = 4.065, p < 0.001
15-33 deg: t(18) = 4.567, p < 0.001
33-88 deg: t(18) = 3.779, p = 0.001

R1.3 There is no direct evidence provided of amygdala feedback but inferred from the observation from the visual cortex alone, which leads to a highly speculative Discussion section. However, the data acquired may allow providing additional and more direct evidence. For example, from the whole brain 3T and 7T BOLD data, a trial-to-trial analysis can be performed to test whether amygdala activity and the claimed diffuse-feedback activity do actually covary, which they have to do according to this hypothesis. Please reanalyze the data with this perspective and present the results in the main manuscript.

Response: We thank the reviewer for this excellent suggestion. We were able to address this comment by performing the analysis that the reviewer described (**new Figure 2**). In this new analysis, we computed an inter-area correlation analysis on both the 3T and 7T BOLD data, analyzing the residual time series after regressing out the task-related component. We found that facial valence affects the functional connectivity between the amygdala and almost all visual cortical areas, including both central V1 where the face stimuli were presented, and peripheral V1, beyond the spatial extent of the face stimuli. This result is consistent with anatomical evidence of diffuse feedback projections from basal amygdala to a number of visual areas, including TE and V1 in monkeys[4,5]. In contrast, there was no functional connectivity valence effect between V1 and any other cortical area, including V2 and V3. This observation suggests that intracortical feedback within visual cortex is unlikely to explain the valence effects that we report in the manuscript.

Page 7-9, Results:
"Correlation between amygdala and visual cortex enhanced by fearful faces
We performed an inter-area correlation analysis to test whether the widespread valence effect (Fig. 1b-c) is due to input from the amygdala, or, alternatively, to pervasive cortico-cortical interactions. We characterized changes in intrinsic activity fluctuations that were not directly induced by the stimulus[6] by removing (i.e., regressing out) the stimulus-driven component of the fMRI BOLD time series (Fig. 2a, orange line) from the measured response time series (Fig. 2a, green line) averaged across voxels within each ROI. This procedure produced a residual time series (Fig. 2a, purple line), separately for each ROI and for each participant, that were then used to construct correlation matrices between each pair of ROIs. Two matrices were constructed: one matrix corresponding to epochs of fearful faces, and the other corresponding to epochs of neutral faces. Finally, we computed the valence effect by subtracting the neutral correlation matrix (Fig. 2b, middle) from the fearful correlation matrix (Fig. 2b, left). If valence information reaches V1 via intracortical feedback projections, intrinsic fluctuations between V1 and adjacent extrastriate areas, such as V2 or V3, should be higher in the fearful than in the neutral condition. In contrast, if valence information reaches V1 via direct anatomical projections from the basal amygdala, the intrinsic fluctuations between V1 and the amygdala should be higher in the fearful than in the neutral face condition.

We found that all visual areas are highly and positively correlated with one another during viewing of both fearful and neutral faces (Fig. 2b, left and middle). It is important to note that these strong correlations were not a result of stimulus-evoked responses, since they were regressed out of the measured time series. Instead, these strong correlations likely reflect connectivity among visual cortical areas[7]. We also observed positive correlations between the amygdala and the rest of visual cortex (Fig. 2b, left and middle, 1st column), though amygdala-cortical correlations were substantially lower than cortico-cortical correlations. Finally, the correlation differences between fearful and neutral face conditions (i.e., the valence effect) were evident between the amygdala and almost all visual cortical areas (Fig. 2b, right, 1st column), consistent with findings of diffuse feedback-like projections from basal amygdala to a number of visual areas, including V1 in monkeys[4,5]. In contrast, inter-area correlation valence effect was not evident between V1 and any other cortical area, including V2 or V3 (Fig. 2b, right, 2nd-3rd columns), suggesting that intracortical feedback is unlikely the source of the valence effect in V1.

Next, we tested the retinotopic specificity of the inter-area correlation valence effect. Functional imaging, brain stimulation and behavioral results suggest that feedback projections from ventral cortical areas project to the foveal confluence of early visual cortex[8–10]. In contrast, anatomical projections from the amygdala to V1 are retinotopically diffuse, and project widely throughout V1[4]. Hence, if the valence effect in V1 were due to communication with other visual cortical areas, we would expect to observe enhanced correlations only at the fovea. In contrast, if it is due to feedback from the amygdala, we would expect diffuse correlation enhancements, evident at both the fovea and periphery. We constructed a peripheral V1 ROI, extending from beyond the stimulus representation all the way out to 88 deg of visual angle. We observed robust correlation enhancements even in the peripheral V1 (Fig. 2b, right, 3rd column), consistent with diffuse feedback projections from basal amygdala to V1."

Page 25, Methods:

*"**Inter-area correlation analysis***

The goal of this analysis was to quantify the strength of correlations between brain areas using the component of the time series that was not driven by the task or the stimulus. To remove the stimulus-related component of the BOLD time series, we computed the residual time series after removing the mean stimulus-evoked responses. Mean stimulus-evoked responses were estimated using deconvolution[11], separately for each ROI, in each scan session (see Fig. 2a for one run from an example participant). Specifically, a predicted time series $\hat{y}$ was computed by multiplying the design matrix by the parameter estimates $\hat{x}$. Next, the residual time series was computed by subtracting the predicted response time series from the measured response time series, $r = y - \hat{y}$. Epochs of residual time series (each face block and its following fixation block) corresponding to each facial expression condition (fearful, neutral, happy) were concatenated across runs within a scan session and extracted for the inter-area correlation analysis.

Correlation coefficients between each pair of ROIs (defined above) were computed from the residual time series in each ROI corresponding to each facial expressions condition. The differences (fearful − neutral) in correlations were also computed (Fig. 2b). For participants who were scanned in multiple sessions, correlation coefficients were averaged between sessions."

R1.4 To obtain cortical depth profile, the data is upsampled by 4 and then 21 cortical depths calculated over the ROIs. However, looking at Fig 2c and 2e, the number of voxels included in the VASO analysis seems to be low, questioning whether it is feasible to determine cortical depths in sufficient detail. Upsampling data to a higher resolution does not increase the information content of data. Please provide the number of original voxels in each subject included in the cortical depth analysis in a Table and discuss the potential impact of these numbers on the results.

Response: The reviewer raises a fundamental point: how is it possible to make inferences regarding cortical depth when the functional measurements are coarse relative to the spatial scale of the cortical thickness. Indeed, V1 is, on average, 2.5 mm thick, while the sampling resolution of the VASO measurements are 0.8x0.8x0.8 mm. This means that there are, at most, three voxels extending from the white matter to the pial surface. This consideration might suggest that it makes little sense to define 21 cortical depths. However, the story is more complex. Because of the convoluted shape of the cortical ribbon, a single voxel can reflect activity from a range of cortical depths. A recommended analysis pipeline adopted by the field (e.g., see https://layerfmri.com/2019/02/22/how-many-layers-should-i-reconstruct/#more-1330) is to first upsample the volumetric time series data in each direction, and then average the signal within each anatomically-defined cortical depth (which, in our case, is the VASO-derived T1-weighted anatomy). This procedure of "upsampling followed by averaging" is thought to produce the most accurate estimate of layer-specific activity, though it is important to note that activity in consecutive layers is not statistically independent (i.e., the layer profiles are smooth). In the **new Supplementary Table 1**, we provide the number of voxels in each of the 21 layers in each 7T VASO scan (number of unique participants = 10, total scan sessions = 15). Note that the increase in number of voxel counts with layer is a known feature of the layer generation process and is due to the geometry of the cortex (the outer-most layers simply have more surface area). Also, please note that these are voxel counts are in the upsampled resolution (x4). Because the layers are defined on this fine grid, it is not possible to compute the number of voxels per layer at the original resolution.

R1.5 Together with the VASO data, the BOLD signal was acquired with high spatial resolution. Please show the cortical depth profile for the same contrasts also using the BOLD signal and discuss commonalities and discrepancies. It is also written that: “By measuring CBV responses across cortical layers (Fig. 2c-e), our approach enabled layer-specific measurements of V1 activity that are inaccessible with BOLD fMRI.” This is a strange statement as most cortical depth fMRI studies are done using the BOLD contrast. Please explain why the authors think that BOLD fMRI is not useful for layer-specific studies.

Response: We agree with the reviewer's assessment that the vast majority of layer-specific fMRI studies have been performed with BOLD contrast. However, there is growing appreciation of the fact that BOLD fMRI measurements are contaminated by the macrovasculature, which has a laminar bias[12]. The confounding effects of the macrovasculature are complex and sometimes difficult to interpret. For example, a number of studies have reported a large and nearly linear increase in BOLD response amplitude from deep to superficial layers, and it is thought that this vascular effect could swamp out layer-specific differences in neural activity. VASO has worse signal-to-noise than BOLD, but is thought to not be contaminated by the effects of large draining veins[13]. Below we show the cortical depth profile for each facial expression using both the BOLD (left) and VASO (right) measurements. There is a monotonic-like increase in BOLD signals from deep to superficial layers (peak at the superficial cortical depth), which is qualitatively different from layer profile in the VASO response where the peak is evident nearer the middle layers. VASO is now an established tool in the fMRI literature, but has been mostly used to study frontal lobe areas (motor cortex[14] and prefrontal cortex[15]). Our observation that VASO overcomes the effects of draining veins in visual cortex is relatively novel, but recently confirmed by Akbari et al. (2021)[16].

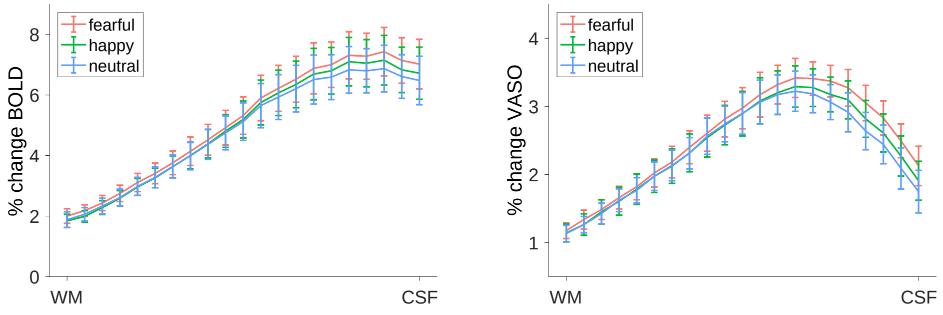

In the revised manuscript, we modify this sentence on **Page 9, Results**:

"By measuring CBV responses across cortical layers (Fig. 3c-e), our approach enabled layer-specific measurements of both feedforward and feedback activity in V1 and overcame the potential confounds introduced by draining veins that are inherent to BOLD fMRI[16]."

R1.6 The authors demonstrate that gender judgment task and face valence are correlated but dismiss the possibility that the gender task may (partially) explain the results: "An RSA on pixel-level discriminability between female and male faces in each expression group revealed a significant effect of expression on gender discriminability (one-way ANOVA: all F values > 148.03, all P values < 0.001 across Euclidian distance, correlation distance and cosine distance)." And: "… gender judgement task on the face stimuli, unrelated to facial expression". Please clarify what "unrelated" means in this context and provide statistical maps and results related to gender judgment (both for cortical depth and regional analysis).

Response: We had subjects perform a gender judgement task on each face stimulus (appearing at about 1 Hz) in order to divert their attention from the emotional expression of each face, which changed at a much slower rate (~ 20 s blocks for each expression). Like the reviewer, we are also interested in whether there are reliable fMRI responses according to gender judgment. However, because the face stimuli updated at such a fast rate, it is impossible to analyze the fMRI data to test for such an effect.

R1.7 The experimental design is suboptimal. The face blocks are twice as long in duration compared to the fixation blocks. Usually, the rest or control duration in fMRI studies is longer than the stimulus duration to allow the hemodynamic signals return to baseline, due to adaptation, post-stimulus inhibition and undershoot etc. Thus, the short fixation duration may influence the value of the baseline signal, which itself may have cortical depth specific effects. Ideally new data has to be acquired to rule out such possibility. Alternatively, the authors should discuss this issue in the Discussion section.

Response: We respectfully disagree. Because our goal was to contrast the different conditions (i.e., fearful vs. neutral facial expressions), using shorter 'blank' blocks than the stimulus blocks was appropriate. In fact, a two-condition block design (alternating blocks of fearful and neutral, with no fixation blocks) would have been even better. We were able to estimate stable response amplitudes (relative to baseline) because the order of the different conditions (fearful, neural, happy) was fully randomized. Had our goal been to estimate the time course of the hemodynamic response, it would have been better to vary the duration of the fixation blocks (i.e., in an event-related protocol), but this was not our goal.

Minor:

R1.8 Fig. 2d. What is “Response phase”? Please clarify.

Response: We used a correlation analysis[17] in which we report the amplitude and phase of the best-fitting sinusoid. In this analysis, the phase indicates the timing of the response. Relatively uniform phases also indicate good signal-to-noise ratios. We have clarified how to interpret response phase in the revision.

Page 9, Results
“Although VASO measurements typically have lower signal-to-noise ratios than BOLD, they are less contaminated by high-amplitude responses in superficial layers due to large draining veins[13] (Fig. 3c). Finally, we note that VASO responses have the opposite sign from BOLD responses, as was evident in the 180° shift in the response phase, indicating that the VASO responses reached a minimum at roughly the same point in time in which BOLD responses reached their maximum (Fig. 3d). This observation indicates that the VASO pulse sequence that we used was indeed sensitive to CBV, rather than residual BOLD effects[14], which would expect to share the same response phase.”

Reviewer #2 (Remarks to the Author):

The authors investigated the valence effect (VE) in human primary visual cortex using VASO and BOLD fMRI. They derived a measure of VE at a cortical-depth dependent level as well at a retinotopic level. Results indicate a cortical-depth dependent specificity of VE as well as a diffuse retinotopic effect, suggesting that valence information is processed in the amygdala and fed back to early visual cortex.

The main research question is of general interest and revolves around the investigation of feedback mechanisms affecting early visual cortex. From a methodological perspective the investigation is solid, and the data is convincing.

I believe several points should be addressed before granting publication regarding the implications of the experimental design adopted and the interpretation of the results.

Response: We thank the reviewer for the positive assessment of the manuscript, and we agree that the following points raised by the reviewer are essential. In our revised manuscript, we are pleased to perform additional analyses suggested by the reviewer and believe the results have strengthened the paper.

R2.1 Regarding the specificity of the amygdala/V1 pathway: Based on the specific pattern of results observed, the authors conclude that these are compatible with the known anatomical connectivity between the amygdala and V1. I agree with the authors that this might be the most likely pathway behind the observed results. However, the interpretation would be corroborated by also showing more specific link between the activity in the amygdala and V1 in the current data. For example, looking at amygdala-V1 correlations in the current dataset (low-resolution) compared to other areas, as V2-V1 or V3-V1 correlation. This type of question could be addressed at the functional connectivity level or as a correlation of the valence effect measured in the two areas.

Response: We thank the reviewer for this suggestion, which is conceptually similar to Reviewer 1's (see comment #3, above). We have now performed this analysis and included a new figure in the resubmission (**new Figure 2**). In short, we found that inter-area correlations between amygdala and many visual cortex areas were enhanced during viewing of fearful faces. Enhanced correlations between amygdala and V1 were retinotopically diffuse (present beyond the spatial extent of the visual stimuli). In contrast, inter-area correlations between occipital lobe visual areas (V1-V3) were not enhanced, suggesting that intra-cortical feedback was not the driver of enhanced response amplitude in V1. This pattern of results is consistent with the hypothesis that amygdala sends diffuse feedback projections to much of visual cortex and provides a firmer footing for investigating the laminar distribution of these feedback projections.

R2.2 Considering my comment above, I would suggest rephrasing or toning down this point in the discussion (pg. 13) 'Here, we use layer-specific fMRI to isolate a circuit arising from a subcortical area, the amygdala, that plays a powerful role in shaping responses at the earliest stage of visual cortical processing.'

Response: We have rephrased this sentence, which now reads: "Here, we applied layer-specific fMRI to understand how visual cortical response are modulated by fearful faces, and in particular, the role of the amygdala in this process." (Page 15)

R2.3 Alternative hypotheses put forward in the introduction and the discussion: In the introduction two alternative hypotheses are clearly stated, leading to specific pattern of expected results. In the discussion the pattern of results is interpreted with respect to three alternatives, one of which is referred to also in the introduction (the second possibility). This difference between the number of alterative hypotheses in the introduction/discussion could create some confusion and should potentially be homogenised.

Response: We thank the reviewer for pointing our different set of hypotheses in the Introduction vs. Discussion. We agree with the reviewer that it is confusing, so we revised the paragraph below.

Pages 15-16, Discussion
"In addition to the feedforward response we measured, the neural pattern of valence modulation we characterized— functionally correlated between the amygdala and both central and peripheral V1 (Fig. 2), specific to the superficial cortical depths of V1 (Fig. 3), retinotopically non-specific, and evident throughout the spatial extent of V1 (Fig. 4)—suggests that sensitivity to facial valence in V1 arises from direct anatomical projections from the amygdala. ***This pattern is inconsistent with the alternative anatomical pathway we considered in the introduction.*** *That is, valence information computed in the amygdala reaches V1 via cortico-cortical feedback projections from extrastriate areas[18]. Although many visual areas exhibited a valence effect (Fig. 1b-c) and also send feedback projections to V1[19], projections from these areas terminate in both superficial and deep layers[20], inconsistent with the layer profile we observed.* ***The layer-specific and retinotopically diffuse pattern is further inconsistent with two additional alternative pathways we consider.*** *One alterative pathway is the cholinergic projections from the basal forebrain. The basal forebrain receives prominent inputs from the amygdala[21] and also sends projections to V1[22]. However, afferents from basal forebrain to V1 terminate in all layers, and are most dense in layers 1, 4 and 6 in macaque[23], making this pathway an unlikely candidate to explain our fMRI results. The other possibility is that the valence information is computed in the pulvinar[24], not in the amygdala, and this information is then transmitted to V1. Pulvinar afferents are mainly located in layer I of V1 in primates[25], consistent with our layer fMRI results. However, these pulvinar-V1 projections are retinotopically specific[26], and would not produce the diffuse*

pattern of valence modulation that we observed. We therefore conclude that direct projections from the amygdala are the most likely source of valence modulation in V1."

R2.3 Positive valence: For completeness of the analysis, I would suggest also reporting the results from the positive valence – neutral controls along depth. While these results are partially described in supplementary figure 4, I would dedicate a section about positive valence effect along cortical depth.

Response: For the sake of completeness, we have now included a layer profile for all three conditions (fearful, happy, and neutral) and included this plot in **new Supplementary Figure 5**. This new figure makes clear that there was very little—if any—modulation with happy faces. We feel that the difference between positive and negative valence is interesting, but constitutes a separate topic that we are currently studying with other experiments.

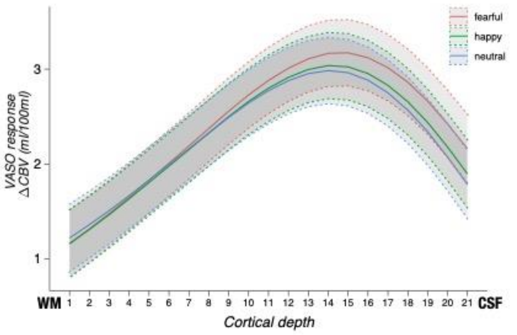

Supplementary Figure 5 | Percent change in VASO (in units of ml per 100 ml CBV) to each facial expression (fearful, neutral, and happy) as a function of relative cortical depth between WM (left) and CSF (right). Red line, fearful; green line, happy; blue line, neutral. The shaded bands bounded with dotted lines indicate the uncertainty range of one standard error for the three fitted profiles. Number of unique participants scanned at 7T VASO: *n*=10 (15 scan sessions, see Table 1).

R2.4 Eye movements: Given the negative valence index shows such diverse distribution patterns across so many areas, is there a possibility there are multiple mechanisms at play? For example: eye movements were not monitored online (even if the task was just to fixate), could some of the activity (e.g. FEF) due to different eye movement patterns when presented with neutral faces/fearful faces?

Response: We too were struck by how widely distributed negative valence effects are throughout the brain. However, we were pleased to see that another recent fMRI study has reported similarly widespread effects (Bo et al., 2021)[18]. Indeed, we think it is likely that there are multiple mechanisms involved in processing affective stimuli (e.g., changes in perceptual processing, arousal, memory, motor output). Regarding eye movements, there are at least two conceivable ways in which oculomotor considerations could have interacted with our results, though we think neither is very likely (**see Discussion on p. 14 of the revised manuscript**).

First, while subjects were instructed to fixate throughout the entire experiment, they inevitably made microsaccades while fixating, and it is conceivable that microsaccade rate and/or direction were modulated by facial valance. But it is difficult to see how changes in microsaccades could have produced the pattern of activity that we observed. Each microsaccade would cause some degree of retinal stimulation when stable visible features (e.g., the stimulus or the edge of the screen) move across the retina. However, we found that the valence effect extended from 0.5 deg all the way to 88 deg (see **new Figure 4),** well beyond both the stimulus and the screen edge. Moreover, microsaccades would be expected to evoke positive BOLD responses in visual cortex[27], but we found negative responses beyond the stimulus representation (which was most likely due to surround suppression associated with the stimulus).

Second, it is conceivable that fearful faces caused pupil dilation, which would in turn allow more photons to enter the eye, resulting in a global response in visual cortex. The percentage change in pupil size needed to effect such a large change in cortical activity would need to be dramatic[28]. Nonetheless, we think that this possibility is worth considering in future experiments.

R2.5 fMRI contrast: While it is clear that figure 2 shows VASO and 7T BOLD results, the same is

not true for figure 1 and figure 3. Please report in the manuscript or the figure caption the field strength at which the data was collected (7T or 3T).

Response: We have now added a relevant figure caption to each figure describing from which specific scan the results come.

R2.6 Gender judgement: In page 4 the authors define the gender judgement task as demanding. The average accuracy across the three experiments range between 91 and 92%. I think this does not count as a demanding task, as it is almost at ceiling level. I would remove the term 'demanding'.

Response: We agree and have revised the manuscript to remove the term 'demanding'.

R2.7 Supp fig. 7, legend: I think from the legend (in gray) it is very difficult to guess which colour is supposed to correspond to which session, I would suggest reformatting this figure / legend with a more intuitive mapping.

Response: Thank you for pointing this out. In the updated version of this figure (**new Supplementary Figure 8**), we now use three shades of gray and dotted lines to denote different sessions consistently across all participants.

Reviewer #3 (Remarks to the Author):

This study is well described, and uses novel acquisition and painstaking analysis approaches to address an interesting research question. I am unable to assess the reliability of the VASO technique to identify layer-specific activity, but the results are credible, despite the small sample of 10 subjects.

The primary concern I have with this paper is the unqualified premise that V1 is enhanced by emotional cues. The evidence for this is sparse, while the evidence against is at least as strong, if not stronger. Only the Pessoa paper lists V1 as sensitive to emotional quality. In the Lang '98 and Bradley '03 studies cited, there is no specific evidence for emotion effects in V1. These studies used natural scenes and analyses collapsed across entire coronal slices of the brain. Other similar scene studies that employed perceptually balanced emotional and neutral stimuli (Sabatinelli et al., 2009, J Nsci) explicitly test emotion effects and report no difference in BOLD signal in calcarine fissure. This is consistent with fMRI meta analyses of emotional face processing, which have not identified primary visual cortex. The Amaral research suggests strong connectivity from basal amygdala to TE, but weak connectivity with V1. The Yamamori & Rockland paper offers some evidence for thalamocortical connectivity with V1, but only suggestive evidence for amygdala-V1 connectivity. Thus the paper might include some discussion of this possibility in the introduction.

Response: Yes, we agree, valence effects in V1 have indeed been reported in some[18,24,29,30], but not all[31–34], studies. We also agree with the reviewer that the Lang '98 and Bradley '03 studies do not provide the most direct support for our hypotheses. We find the seemingly contradictory nature of this prior literature[18,24,29–34] to be perhaps the strongest motivation for our study. We present the largest sample of V1 responses to emotional stimuli that has ever been reported, across multiple imaging modalities (3T, 7T, BOLD, VASO), and in so doing, provide definitive support for the hypothesis that early visual cortex is sensitive to emotional faces. Our finding of enhanced V1 activity to fearful faces is consistent with behavioral evidence showing that emotional stimuli potentiate the processing of oriented gratings[35,36].

We have updated the references on valence effects in V1 throughout the revised manuscript. We have also expanded our discussion on **Page 14**.

"Third, despite converging evidence of valence sensitivity in early visual cortex from human EEG/ERP[37], recordings in awake monkey[38], and computational modeling[39], fMRI evidence for valence sensitivity in human early visual cortex has been conflicting, with clear effects reported in some[18,24,40], but not all[31,41,42] studies. Our results demonstrate clear and reliable valence sensitivity throughout human visual cortex, including in V1."

Given that Reviewers 1 & 2 felt that a functional connectivity analysis would help support a role for amygdala-V1 feedback in facial valence processing, we have added a major new functional connectivity analysis (**new Figure 2, pages. 7-9 in the main text**) which provides further evidence that V1 (both central and peripheral V1, in line the observations from anterograde tracer studies that amygdala afferents are diffusely distributed throughout V1 in macaque[4,5]) is enhanced by emotional cues.

Other concerns.

R3.1 How might the rapidity of ventral visual cortical activity affect V1 activity relative to the 2+ second sampling rate of fMRI? Could information flow through thalamocortical pathways more rapidly than be accounted for with hemodynamic measures?

Response: As in all fMRI experiments, there is an inherent mismatch between the fast temporal dynamics of neural activity and the sluggishness of hemodynamics (measured with either BOLD or VASO). We overcame this by using a relativity fast visual stimulus presentation (1 Hz), which induces a steady-state hemodynamic response over the entire block of trials. Our analysis targeted the amplitude of the fMRI response integrated over the entire block of trials. We then tested whether this integrated response amplitude differed across conditions (e.g., fearful vs. neutral blocks of trials).

R3.2 Did subjects feel fear during the experiment?

Response: This is an interesting question that we too have wondered about. The amygdala is known to respond not only to fearful-related stimuli, but also to a variety of biologically relevant stimuli, such as animate entities[43], ambiguous or unpredictable cues[44], and social category groups[45], even when subjects do not explicitly report emotional responses. So, in a sense, the logic behind the experiment would hold even if subjects did not feel fear. We now discuss this important issue in the revision.

Pages 15, Discussion.
"Here, we applied layer-specific fMRI to understand visual cortical response are modulated by fearful faces, and in particular, the role of the amygdala in this process. ***Note that amygdala activation may not be specific to fear[46] nor to facial expressions[47]. The amygdala responds to a variety of biologically relevant stimuli, such as animate entities[43], ambiguous or unpredictable cues[44], and social category groups[45]****.*

R3.3 How was gaze controlled or recorded in the MR scanner? Could saccades explain the nonretinotopic effects?

Response: A similar concern was expressed by Reviewer 2 (comment #4). Please see our response above, and the expanded section in the Discussion (**Page 14, Discussion**).

R3.4 Could this be a face-specific effect? As the authors state, the processing of emotional expressions is a multifaceted socio-communicative behavior, distinct from emotion in general.

Response: This is a fundamental question. We do not know whether the facial valence effects that we have characterized here are face-specific, since we only used face stimuli in this experiment. We did observe large modulations in the FFA, suggesting some degree of face specificity. But it would be interesting to test the laminar profile and retinotopic distribution of valence effects with other emotion-evoking stimuli, such as emotional scenes[18]. We now acknowledged that amygdala activation may not be face-specific and extended our discussion about this possibility of stimulus specificity.

Pages 15, Discussion.
"Note that amygdala activation may not be specific to fear[46] nor to facial expressions[47]. The amygdala responds to a variety of biologically relevant stimuli, such as animate entities[43], ambiguous or unpredictable cues[44], and social category groups[45]."

Pages 17, Discussion.
*"The facial valence effect in retinotopic visual cortex we found are broadly consistent with a recent EEG-fMRI study that demonstrated affective scene decoding in retinotopic visual cortex[18]. In that study, however, the amplitude of the late positive potential (LPP)—an index of reentrant processing from the amygdala back to visual cortex[48]—correlated only with the fMRI decoding accuracy in ventral visual cortex, but not in early or dorsal visual cortex, suggesting that the valence effect in early visual cortex may arise from reentrant signals propagated to V1 from ventral visual cortex. This may suggest that valence-related feedback signals are **stimulus specific**, with face stimuli, and perhaps animate objects more generally[43], engaging the circuitry from basal amygdala to V1, and scene stimuli engaging connectivity between ventral visual cortex and V1. Regardless of stimulus type, the valence effect occurs throughout visual cortex in both studies. Thus, future work will need to use network analysis of whole brain dynamics across different imaging modalities to determine whether these widespread valence effects are due to direct influence from the amygdala, or feedforward inputs from V1, or a combination of both."*

References cited in this response letter

1. Chen, G., Taylor, P. A., Cox, R. W. & Pessoa, L. Fighting or embracing multiplicity in neuroimaging? neighborhood leverage versus global calibration. *Neuroimage* **206**, 116320 (2020).
2. Chen, G. *et al.* Beyond linearity in neuroimaging: Capturing nonlinear relationships with application to longitudinal studies. *Neuroimage* **233**, 117891 (2021).
3. Chen, G. *et al.* Handling Multiplicity in Neuroimaging Through Bayesian Lenses with Multilevel Modeling. *Neuroinformatics* **17**, 515–545 (2019).
4. Freese, J. L. & Amaral, D. G. The organization of projections from the amygdala to visual cortical areas TE and V1 in the macaque monkey. *J. Comp. Neurol.* **486**, 295–317 (2005).
5. Freese, J. L. & Amaral, D. G. Synaptic organization of projections from the amygdala to visual cortical areas TE and V1 in the macaque monkey. *J. Comp. Neurol.* **496**, 655–667 (2006).
6. Freeman, J., Donner, T. H. & Heeger, D. J. Inter-area correlations in the ventral visual pathway reflect feature integration. *J. Vis.* **11**, 15–15 (2011).
7. Felleman, D. J. & Van Essen, D. C. Distributed hierachical processing in the primate cerebral cortex. *Cereb. Cortex* **1**, 1–47 (1991).
8. Williams, M. A. *et al.* Feedback of visual object information to foveal retinotopic cortex. *Nat. Neurosci.* **11**, 1439–1445 (2008).
9. Chambers, C. D., Allen, C. P. G., Maizey, L. & Williams, M. A. Is delayed foveal feedback critical for extra-foveal perception? *Cortex* **49**, 327–335 (2013).
10. Fan, X., Wang, L., Shao, H., Kersten, D. & He, S. Temporally flexible feedback signal to foveal cortex for peripheral object recognition. *Proc. Natl. Acad. Sci. U. S. A.* **113**, 11627–11632 (2016).
11. Dale, A. M. Optimal experimental design for event-related fMRI. in *Human Brain Mapping* **8**, 109–114 (1999).
12. Kay, K. *et al.* A critical assessment of data quality and venous effects in sub-millimeter fMRI. *Neuroimage* **189**, 847–869 (2019).
13. Huber, L. *et al.* Slab-selective, BOLD-corrected VASO at 7 tesla provides measures of cerebral blood volume reactivity with high signal-to-noise ratio. *Magn. Reson. Med.* **72**, 137–148 (2014).
14. Huber, L. *et al.* High-Resolution CBV-fMRI Allows Mapping of Laminar Activity and Connectivity of Cortical Input and Output in Human M1. *Neuron* **96**, 1253–1263 (2017).
15. Finn, E. S., Huber, L., Jangraw, D. C., Molfese, P. J. & Bandettini, P. A. Layer-dependent activity in human prefrontal cortex during working memory. *Nat. Neurosci.* **22**, 1687–1695 (2019).
16. Akbari, A., Bollmann, S., Ali, T. S. & Barth, M. Modelling the depth-dependent VASO and BOLD responses in human primary visual cortex. *bioRxiv* 2021.05.07.443052 (2021). doi:10.1101/2021.05.07.443052
17. Freeman, J., Brouwer, G. J., Heeger, D. J. & Merriam, E. P. Orientation decoding depends on maps, not columns. *J. Neurosci.* **31**, 4792–4804 (2011).
18. Bo, K. *et al.* Decoding Neural Representations of Affective Scenes in Retinotopic Visual Cortex. *Cereb. Cortex* **00**, 1–17 (2021).
19. Van Essen, D. C., Anderson, C. H. & Felleman, D. J. Information processing in the primate visual system: An integrated systems perspective. *Science* **255**, 419–423 (1992).
20. Rockland, K. S. & Virga, A. Terminal arbors of individual “Feedback” axons projecting from area V2 to V1 in the macaque monkey: A study using immunohistochemistry of anterogradely transported Phaseolus vulgaris-leucoagglutinin. *J. Comp. Neurol.* **285**, 54–72 (1989).

21. Russchen, F. T., Amaral, D. G. & Price, J. L. The afferent connections of the substantia innominata in the monkey, Macaca fascicularis. *J. Comp. Neurol.* **242**, 1–27 (1985).
22. Lean, G. A., Liu, Y. J. & Lyon, D. C. Cell type specific tracing of the subcortical input to primary visual cortex from the basal forebrain. *J. Comp. Neurol.* **527**, 589–599 (2019).
23. Hedreen, J. C., Uhl, G. R., Bacon, S. J., Fambrough, D. M. & Price, D. L. Acetylcholinesterase-immunoreactive axonal network in monkey visual cortex. *J. Comp. Neurol.* **226**, 246–254 (1984).
24. Koizumi, A. *et al.* Threat anticipation in pulvinar and in superficial layers of primary visual cortex (V1). Evidence from layer-specific ultra-high field 7T fMRI. *eNeuro* **6**, (2019).
25. Shipp, S. The functional logic of cortico-pulvinar connections. *Philosophical Transactions of the Royal Society B: Biological Sciences* **358**, 1605–1624 (2003).
26. Purushothaman, G., Marion, R., Li, K. & Casagrande, V. A. Gating and control of primary visual cortex by pulvinar. *Nat. Neurosci.* (2012). doi:10.1038/nn.3106
27. Tse, P. U., Baumgartner, F. J. & Greenlee, M. W. Event-related functional MRI of cortical activity evoked by microsaccades, small visually-guided saccades, and eyeblinks in human visual cortex. *Neuroimage* **49**, 805–816 (2010).
28. Becket Ebitz, R. & Moore, T. Both a gauge and a filter: Cognitive modulations of pupil size. *Frontiers in Neurology* **10**, 1190 (2019).
29. Pessoa, L., McKenna, M., Gutierrez, E. & Ungerleider, L. G. Neural processing of emotional faces requires attention. *Proc. Natl. Acad. Sci. U. S. A.* **99**, 11458–11463 (2002).
30. Lang, P. J., Fitzsimmons, J. R., Bradley, M. M., Cuthbert, B. N. & Scott, J. Processing emotional pictures: Differential activation in primary visual cortex. *Neuroimage* **3**, S231 (1996).
31. Sabatinelli, D., Lang, P. J., Bradley, M. M., Costa, V. D. & Keil, A. The timing of emotional discrimination in human amygdala and ventral visual cortex. *J. Neurosci.* **29**, 14864–14868 (2009).
32. Bradley, M. M. *et al.* Activation of the visual cortex in motivated attention. *Behav. Neurosci.* **117**, 369–380 (2003).
33. Sabatinelli, D. *et al.* Emotional perception: Meta-analyses of face and natural scene processing. *NeuroImage* **54**, 2524–2533 (2011).
34. Lang, P. J. *et al.* Emotional arousal and activation of the visual cortex: An fMRI analysis. *Psychophysiology* **35**, 199–210 (1998).
35. Phelps, E. A., Ling, S. & Carrasco, M. Emotion facilitates perception and potentiates the perceptual benefits of attention. *Psychol. Sci.* **17**, 292–299 (2006).
36. Barbot, A. & Carrasco, M. Emotion and anxiety potentiate the way attention alters visual appearance. *Sci. Rep.* **8**, 1–10 (2018).
37. Thigpen, N. N., Bartsch, F. & Keil, A. The malleability of emotional perception: Short-term plasticity in retinotopic neurons accompanies the formation of perceptual biases to threat. *J. Exp. Psychol. Gen.* **146**, 464–471 (2017).
38. Li, Z., Yan, A., Guo, K. & Li, W. Fear-Related Signals in the Primary Visual Cortex. *Curr. Biol.* **29**, 4078-4083.e2 (2019).
39. Kragel, P. A., Reddan, M. C., LaBar, K. S. & Wager, T. D. Emotion schemas are embedded in the human visual system. *Sci. Adv.* **5**, (2019).
40. Pessoa, L. & Adolphs, R. Emotion processing and the amygdala: From a 'low road' to 'many roads' of evaluating biological significance. *Nature Reviews Neuroscience* **11**, 773–782 (2010).
41. Lang, P. J. *et al.* Emotional arousal and activation of the visual cortex: An fMRI analysis. *Psychophysiology* **35**, 199–210 (1998).
42. Bradley, M. M. *et al.* Activation of the visual cortex in motivated attention. *Behav. Neurosci.* **117**, 369–380 (2003).

43. Yang, J., Bellgowan, P. S. F. & Martin, A. Threat, domain-specificity and the human amygdala. *Neuropsychologia* **50**, 2566–2572 (2012).
44. Davis, F. C., Neta, M., Kim, M. J., Moran, J. M. & Whalen, P. J. Interpreting ambiguous social cues in unpredictable contexts. *Soc. Cogn. Affect. Neurosci.* **11**, 775–782 (2016).
45. Freeman, J. B., Schiller, D., Rule, N. O. & Ambady, N. The neural origins of superficial and individuated judgments about ingroup and outgroup members. *Hum. Brain Mapp.* **31**, 150–159 (2010).
46. Fitzgerald, D. A., Angstadt, M., Jelsone, L. M., Nathan, P. J. & Phan, K. L. Beyond threat: Amygdala reactivity across multiple expressions of facial affect. *Neuroimage* **30**, 1441–1448 (2006).
47. Britton, J. C., Taylor, S. F., Sudheimer, K. D. & Liberzon, I. Facial expressions and complex IAPS pictures: Common and differential networks. *Neuroimage* **31**, 906–919 (2006).
48. Liu, Y., Huang, H., McGinnis-Deweese, M., Keil, A. & Ding, M. Neural substrate of the late positive potential in emotional processing. *J. Neurosci.* **32**, 14563–14572 (2012).
49. Callaway, E. M. Local circuits in primary visual cortex of the macaque monkey. *Annual Review of Neuroscience* **21**, 47–74 (1998).
50. Douglas, R. J., Martin, K. A. C. & Whitteridge, D. A Canonical Microcircuit for Neocortex. *Neural Comput.* **1**, 480–488 (1989).
51. Yamamori, T. & Rockland, K. S. Neocortical areas, layers, connections, and gene expression. *Neurosci. Res.* **55**, 11–27 (2006).
52. Benson, N. C., Butt, O. H., Brainard, D. H. & Aguirre, G. K. Correction of Distortion in Flattened Representations of the Cortical Surface Allows Prediction of V1-V3 Functional Organization from Anatomy. *PLoS Comput. Biol.* **10**, e1003538 (2014).
53. Huber, L. (Renzo) *et al.* LAYNII: A software suite for layer-fMRI. *bioRxiv* 2020.06.12.148080 (2020). doi:10.1101/2020.06.12.148080

Reviewers' comments:

Reviewer #1 (Remarks to the Author):

The authors have answered all my points and the manuscript improved a lot. However, there are still fundamental problems with the paper, which have not been addressed by the responses.

1. The authors have performed Bayesian Multilevel (BML) modeling. However, this analysis seems to obscure more than solve the underlying problems. They say that this addresses subject-level variability. What does this mean? Variability between or within subjects? If the latter: over trials, spatially or both? Given the very small size of the effect, it is hard to believe that the authors did account for all sources or variability and error. For example, in Fig 3.f the maximum is around 3ml/100ml and in Fig 3.g the difference between fearful and neutral faces is around 0.0035 ml/100ml, which is 0.0035/3 ~ 0.001 or 0.1%. Even if the effect would be much larger (say in the few % range) and given the noise level of laminar VASO, this is certainly beyond the level of sensitivity of VASO to detect effects (see also #5). Even if it were significant, strong conclusions of such a small irrelevant effect size is not warranted. The effect size is also small for the low resolution BOLD data (e.g. valence effect is around 0.08 in correlation coefficient). Please clarify and comment in the manuscript.
2. The authors added a functional connectivity analysis using the residuals of the BOLD time courses after removing the stimulus effects. To determine the stimulus response, a canonical hemodynamic response functon(HRF) is used. However, how do the authors know that the HRF is accurate and the same HRF applicable for all brain areas? Any error in the HRF will results in wrong residuals (see also below #3) and hence functional connectivity results. In addition, in Fig. 2., the residual and original time courses in amygdala are almost identical. Does this mean that there is no stimulus response in the amygdala? Finally, it is known that amygdala is close to a big vein, which partially drains the visual cortex and is sensitive to systemic effects. How did the authors make sure that systemic and draining vein effects do not play a role? Please discuss the issue of HRF, biases and residuals in more depth.
3. My previous concern on the length of the rest periods was dismissed by the authors as they argue that they are not interested in the HRF shape, which is not acceptable as the residuals and, hence, some of the main conclusions depend on the exact shape of the HRF. As can be seen in Fig 2.a., the BOLD signal level between the blocks is lower than the initial baseline, indicating a strong presence of the post-stimulus undershoot. That is, the previous block influences the BOLD signal level of the next block. Given that the effect sizes are small and the short duration of each run, this is a serious flaw of the experimental design. This can only be resolved by acquiring new data with rest periods at least twice as long as the stimulus block durations.
4. In the previous review, it was asked that the authors provide the number of original voxels. Now, the authors provide number of voxels but in the upsampled resolution in suppl material, arguing that it is not possible to provide this number in the original resolution. I do not understand this: the authors know the approximate location of the ROIs, which they should be able to project into the original functional space and then this number should be easily be extractable.
5. Even though the methodological question what VASO represents is beyond the current study, it is recommended to provide a more balanced view. The laminar fMRI community acknowledges that the BOLD signal suffers from draining vein bias. However, increasingly there is more skepticism whether VASO really reflects CBV: a) as the authors show the VASO response is ~3% of tissue volume, which is factor of 5-20 too large for a physiological CBV change (i.e. CBV changes ~20% of baseline CBV for a strong stimulation; for a baseline CBV of 3%, this corresponds to 0.6% relative to tissue volume and, as the stimuli evoke a small BOLD response, the expected CBV change also has to be smaller, probably in the range of 0.1-0.2 ml/100ml). In addition, the BOLD contamination of VASO is only removed under ideal conditions. Given the small effect size, this BOLD contamination can be a factor in the presented VASO data and conclusions. Please discuss these issues in a balanced way.

Reviewer #2 (Remarks to the Author):

The authors included a new functional connectivity analysis from the 7T BOLD experiment. The analysis is based on the residuals of the responses for each area in visual cortex and the amygdala.

1.1
Based on figure 2, the correlation coefficient between the visual hierarchy and amygdala (V1, V2 etc etc) appears to be small (black to dark grey color) for the fearful and neutral conditions.
The authors compute the difference in connectivity between fearful and neutral condition, reporting that the difference in correlation is significantly different than zero between the amygdala and visual cortex.
Difference in connectivity is a derivative measure. I do not have a problem with derivative measures per se, but in this case the starting points (correlation in the fearful and correlation in neutral conditions) are very small (close to zero), indicating in essence no connectivity. We are in presence of two measures that indicate no connectivity, whose difference led to a measure that is interpreted as significant connectivity.
I am not entirely convinced by this logic. If we were starting from two significant measures, I would have less problems in taking the difference between the two and interpreting the result.

1.2
The observation of low connectivity between V1 (early visual cortex in general) and the amygdala is consistent with the remark made by Reviewer 3 (and the literature).

1.3
Are the p values from the Wilcoxon signed rank tests reported in figure 2 corrected for the number of comparisons?

To summarize, I am not convinced that this analysis shows corroborating evidence of functional connectivity between the amygdala and V1.

2. Positive Valence effect.
The cortical depth dependent effect for happy faces is now reported (to a limited degree) in the supplementary material. I still believe that a full description of the valence effect for positive faces is necessary to complement the results for fearful faces. For example, it could give insights about the specificity of the cortical depth-dependent results regarding the emotion and the connotation (positive or negative) conveyed.
Moreover, given that the experiment included 3 conditions: fearful, neutral (control) and happy, a reader would naturally expect to see the results from the happy condition as well.

Reviewer #3 (Remarks to the Author):

I believe the authors have revised their work extensively and convincingly, and that this paper will make a solid contribution to the literature. I have 1 issue that could be addressed with minor language revisions.

My only concern is the conflation of face and scene perception in lines 346-351. In this third point, the human studies of scene processing are compared to the current results, under the umbrella concept of valence sensitivity. Please specify which studies used faces, and which used scenes. This is an important difference, as face processing is likely to be supported by distinct mechanisms relative to scenes, as you discuss in the sentences before and after this section, and specifically in lines 465-468.

We thank the reviewers for their insightful comments and suggestions regarding the revision of our manuscript. The reviewers raised a number of issues, the two most serious of which concerned the validity of the experimental design (see R1.3) and the magnitude of correlations between the amygdala and V1 (see R2.1). Below, we provide a point-by-point response to each of their comments, focusing most extensively on these two points. The reviewer's comments are indicated by blue font, below.

Reviewer 1:
The authors have answered all my points and the manuscript improved a lot. However, there are still fundamental problems with the paper, which have not been addressed by the responses.

Major points:

R1.1 The authors have performed Bayesian Multilevel (BML) modeling. However, this analysis seems to obscure more than solve the underlying problems. They say that this addresses subject-level variability. What does this mean? Variability between or within subjects? If the latter: over trials, spatially or both? Given the very small size of the effect, it is hard to believe that the authors did account for all sources or variability and error. For example, in Fig 3.f the maximum is around 3ml/100ml and in Fig 3.g the difference between fearful and neutral faces is around 0.0035 ml/100ml, which is 0.0035/3 ~ 0.001 or 0.1%. Even if the effect would be much larger (say in the few % range) and given the noise level of laminar VASO, this is certainly beyond the level of sensitivity of VASO to detect effects (see also #5). Even if it were significant, strong conclusions of such a small irrelevant effect size is not warranted. The effect size is also small for the low resolution BOLD data (e.g. valence effect is around 0.08 in correlation coefficient). Please clarify and comment in the manuscript.

This comment is related to the Reviewer's comment on the initial submission (quoted below):

(previous review) The effect sizes shown in Fig. 2g and 3c, on which the main conclusion rely upon, are quite small. It is claimed that these are statistically significant. However, the authors test significance on the mean values within subjects, which are assumed to not have any error. In other words, the values shown in these Figures have errors themselves and any statistical test has to use standard error propagation laws. Given, for example, the high spatial variability shown in Fig 3b, it is reasonable to expect that the mean values for each subject in Figure 3c have large errors, which the statistical tests have to take into account. Please recalculate the statistical significance both for cortical depth and regional analyses.

Response: In the first round of review, R1 was concerned about the propagation of within-subject variance in the random-effect analyses that we initially employed. While random-effect analyses are common in the field, we agreed with R1's assessment, specifically with regard to variability across trials within individual subjects. To address this concern in the first revision, we adopted a more sophisticated statistical approach based on Bayesian multilevel (BML) modeling, in which within-subject variability constituted a level of the model. The reviewer now questions whether this approach really incorporates within-subject variance. As we explain below, it does.

The details of the BML can be found in our co-author's recent publications[1–3]. To help clarify how within-subject variance is dealt with, we provide the model formulation using the cerebral blood volume (CBV) data as an example (**new Fig. 3h**):

$y_{ijk} \sim T(\mu_{ij}, \tau_{ij}^2 + \hat{\sigma}_{ijk}^2;\ \eta),$
$\mu_{ij} = \ \alpha_i + \ \theta_j,$
$\theta_j \sim T(0,\ \pi^2;\ \upsilon).$

Here y_{ijk} is the ΔCBV value of subject j at layer i during session k ($i = 1, 2, \ldots, 10$; j = 1, 2, 3; k = 1, 2) that is assumed to follow a Student's *t*-distribution with its variance composed of two sources (η is the number of degrees of freedom): the variability of subject j at layer i, and measurement error $\hat{\sigma}_{ijk}^2$ based on the regression model at the subject level. The effect μ_{ij} of subject j at layer i is assumed to be composed of the effect α_i at layer i and subject-specific effect θ_j that is supposed to follow a Student's *t*-distribution with variance π^2 and υ degrees of free. The incorporation of the term $\hat{\sigma}_{ijk}^2$ in the model above is intended to address the reviewer's previous concern regarding error propagation. As seen in the hierarchical structure of the model formulation, the incorporation of within-subject variability does not mean that the measurement error would be superimposed to the uncertainty of the population-level effects, such as the layer-level effects α_i. Rather, the incorporation of measurement errors more accurately characterizes the hierarchical information and inter-subject relationships, playing a role in weighting among subjects. Such a role of weighting and accuracy improvement is similar to that in the conventional meta-analysis in which study-level measurement errors are carried over to the overall assessment; see Ref[4] for example. We note that the models for other data hierarchical structures are similarly formulated to the one shown above.

R1 drew attention to the small effect size in **Fig. 3g**. The values on the X-axis were erroneously reported in the previous submission. They were off by a factor of 100 (this was a ***typographical error*** in the figure). The effect size was correctly reported in the text in both the initial submission and the revision (see **Fig. 2g** in the initial submission). This was a mistake, and it has now been corrected in **new Fig. 3g**.

Finally, R1 points out that the BML is complicated and hence potentially obfuscates the underlying effects. There is an unavoidable tradeoff here. The random-effects analysis is commonplace, accessible, but makes simplifying assumptions regarding within-subject variance. The BML is complicated, less familiar, but statistically sophisticated. Critically, our results are significant and robust, regardless of which of these two analysis methods we use.

R1.2 The authors added a functional connectivity analysis using the residuals of the BOLD time courses after removing the stimulus effects. To determine the stimulus response, a canonical hemodynamic response functon(HRF) is used. However, how do the authors know that the HRF is accurate and the same HRF applicable for all brain areas? Any error in the HRF will results in wrong residuals (see also below #3) and hence functional connectivity results. In addition, in Fig. 2., the residual and original time courses in amygdala are almost identical. Does this mean that there is no stimulus response in the amygdala? Finally, it is known that amygdala is close to a big vein, which partially drains the visual cortex and is sensitive to systemic effects. How did the authors make sure that systemic and draining vein effects do not play a role? Please discuss the issue of HRF, biases and residuals in more depth.

Response: We would like to clarify that ***we did not use a canonical HRF*** in this functional connectivity analysis. Instead, we used an ***inter-area correlation analysis***[5] to regress out any stimulus or task-evoked response using linear deconvolution, independently allowing for different response shapes in each voxel. We have clarified this important detail in the revision

and have also included a link to the analysis code on github (https://github.com/tinaliutong/layerfmri_AMG_V1/blob/main/analysisCode_Fig.2.m).

The reviewer points out that visually-driven responses in the amygdala are small, and are considerably smaller than those in visual cortex, such as V1. This is indeed the case in our data, and it is also consistent with many previous observations in monkeys and in humans. Single-unit recording studies have reported a much lower spike rate in the amygdala compared to visual cortex[6,7]. Moreover, previous fMRI studies have shown substantially smaller BOLD responses in the amygdala relative to V1[8]. We now note this observation in the text and reference the relevant literature (p. 8, line 163-170):

> *We also observed significant positive correlations between the amygdala and the rest of visual cortex (Fig. 2b-c, left and middle, 1st column), though amygdala-cortical correlations were substantially lower than cortico-cortical correlations. The lower amygdala-cortical correlations could be due to considerably smaller response amplitude in the amygdala (relatively to visual cortex), which has been observed in both human and monkey studies[6,7]. The generally small amygdala-cortical correlations could also reflect signal contamination from a nearby vein[9], large physiological noise in the amygdala, or a combination of both factors.*

The reviewer asked how we can make sure that systemic and draining vein effects do not play a role. We are aware of the large blood vessel proximal to the amygdala (the basal vein of Rosenthal) and the controversy surrounding it[9]. This is a complex issue that the field as a whole, and fMRI studies of the amygdala in particular, have yet to fully contend with. In terms of V1, the layer profile that we measured decreased at the most superficial cortical depths where the vascular effects are expected to be strongest (**Fig. 3f**), suggesting that the issue of draining veins is unlikely to account for the observed layer profile.

R1.3 My previous concern on the length of the rest periods was dismissed by the authors as they argue that they are not interested in the HRF shape, which is not acceptable as the residuals and, hence, some of the main conclusions depend on the exact shape of the HRF. As can be seen in Fig 2.a., the BOLD signal level between the blocks is lower than the initial baseline, indicating a strong presence of the post-stimulus undershoot. That is, the previous block influences the BOLD signal level of the next block. Given that the effect sizes are small and the short duration of each run, this is a serious flaw of the experimental design. This can only be resolved by acquiring new data with rest periods at least twice as long as the stimulus block durations.

This comment is related to the Reviewers comment on the initial submission (quoted below):

(previous review) The experimental design is suboptimal. The face blocks are twice as long in duration compared to the fixation blocks. Usually, the rest or control duration in fMRI studies is longer than the stimulus duration to allow the hemodynamic signals return to baseline, due to adaptation, post-stimulus inhibition and undershoot etc. Thus, the short fixation duration may influence the value of the baseline signal, which itself may have cortical depth specific effects. Ideally new data has to be acquired to rule out such possibility. Alternatively, the authors should discuss this issue in the Discussion section.

Response: With a 9 s fixation block between conditions, the post-stimulus undershoot from one block of trials certainly overlaps with the beginning of the response in the next block of trials.

However, given that the block order is fully randomized, both within and across many runs, we do not believe that this creates any sort of systematic bias, nor does it confound any of the analyses in the manuscript. Nonetheless, the reviewer asked us to either collect new data with longer fixation blocks or discuss this issue in the Discussion. We have now done both. In addition, we have provided a computational simulation to support our claims that this experimental design is not a concern.

New Data: As requested by the reviewer, we have collected *new 7T fMRI data* (see **Supplementary Methods** and **new Supplementary Fig. 9b**) using a range of fixation block durations, starting with the 9 s fixation block that we used in the main experiment, an intermediate-length fixation block (18 s), and with very long fixation blocks (36 s). These new fMRI data confirm our intuition: we see clear facial valence effects in V1 regardless of the duration of the fixation blocks. These new data demonstrate that facial valence effect in V1 is not an artifact of the experimental design.

Expanded Discussion: We have added a section to the Discussion discussing the merits and potential limitations of using short fixation blocks, especially with regard to layer fMRI experiments (see p. 20, line 465-486 of the revised manuscript).

> *Our fMRI experiment employed a block design with three different facial expressions (happy, neutral, fearful) with interleaved fixation blocks that were shorter (half the duration) than the face blocks (Supplementary Fig. 9). With the relatively short fixations block, the post-stimulus undershoot from one face block overlapped with the beginning of the response in the next block of trials. This design is derived from classic experiments in which interleaved fixation blocks were shorter than stimulation blocks (i.e., 30 s stimulus blocks interleaved with 20 s fixation blocks in Kanwisher et al., 1997[10]; 9s stimulation blocks interleaved with 6s blank screen in Levy et al., 2001[11] and Hasson et al., 2002[12]). The fMRI BOLD response approximates a shift-invariant linear system[13], which makes it possible to deconvolve overlapping responses from different conditions, provided the time series is sufficiently long and the conditions sufficiently randomized and counter-balanced[14].*
>
> *There are two important assumptions when applying this design to layer fMRI. The first is that the linearity of the response applies to measurements at each cortical layer. For example, it is conceivable that response at one layer conforms to the linearity assumptions, but responses at other layers deviate from linearity to some degree, perhaps due to directional blood pooling towards the pial surface. Initial studies suggest that linearity assumptions do apply to layer-specific fMRI[15,16], but this issue does deserve greater attention. The second assumption is that the VASO measurements are linear in the same way as BOLD measurements. VASO fMRI is an indirect measurement of CBV, which is thought to exhibit linearity[17]. However, more work on the linearity of VASO is warranted. Nonetheless, slight deviations from linearity, if present in our measurements, are unlikely to account for the results that we report here.*

Computational simulation: To test our intuition regarding the experimental design, we have built a computational "ground truth" simulation in which we simulate responses to happy, fearful and neutral faces using a variety of experimental designs and then attempt to recover the "true" hemodynamic responses (see **Supplementary Methods** and **new Supplementary Fig. 9a**). We find that it is possible to recover the ground truth with the experimental design that we used in our manuscript. A full-blown simulation of the fMRI depth-dependent response is beyond the

scope of this paper. But we hope that these considerations add nuance to our findings and provide the appropriate caveats that the reviewer highlighted.

R1.4. In the previous review, it was asked that the authors provide the number of original voxels. Now, the authors provide number of voxels but in the upsampled resolution in suppl material, arguing that it is not possible to provide this number in the original resolution. I do not understand this: the authors know the approximate location of the ROIs, which they should be able to project into the original functional space and then this number should be easily be extractable.

Response: Indeed, we do know the precise location of the layer-specific ROIs in both the upsampled and original resolution, and we have provided the number of voxels at the original resolution in each 7T VASO scan, as the reviewer requested (new **Supplementary Table 5**), and we have included a description of the voxel counts in the revised manuscript. We note, however, that counting voxels at the original resolution in a layer ROI that is defined on the upsampled grid is somewhat misleading. This is because the cortex is curvy with respect to the voxel grid. This means upsampling and then averaging, as we have done, allow for a weighted average of voxels in the original space (i.e., weighted by the proportion of the voxel's volume that intersects the cortical surface). We describe this issue on pp. 31-32, line 838-844.

> *The number of voxels per layer in the upsampled resolution in each 7T VASO scan is available in Supplementary Table 4. The procedure that we followed, averaging the fMRI response across voxels in a layer ROI that was defined on the upsampled grid, is analogous to taking a weighted average across voxels in the original space (weighted by the proportion of the voxel's volume that intersects the cortical surface, see Supplementary Fig. 10). The number of voxels in the original resolution in each 7T VASO scan is also available in Supplementary Table 5.*

R1.5. Even though the methodological question what VASO represents is beyond the current study, it is recommended to provide a more balanced view. The laminar fMRI community acknowledges that the BOLD signal suffers from draining vein bias. However, increasingly there is more skepticism whether VASO really reflects CBV: a) as the authors show the VASO response is ~3% of tissue volume, which is factor of 5-20 too large for a physiological CBV change (i.e. CBV changes ~20% of baseline CBV for a strong stimulation; for a baseline CBV of 3%, this corresponds to 0.6% relative to tissue volume and, as the stimuli evoke a small BOLD response, the expected CBV change also has to be smaller, probably in the range of 0.1-0.2 ml/100ml). In addition, the BOLD contamination of VASO is only removed under ideal conditions. Given the small effect size, this BOLD contamination can be a factor in the presented VASO data and conclusions. Please discuss these issues in a balanced way.

Response: The VASO response amplitudes that we report are commensurate with those reported by others using the same pulse sequence and scanner at NIH[18,19]. We have added a discussion of the possibility of BOLD contamination, as the reviewer requested (see p. 18, line 414-427). However, we agree that a full methodological treatment of the pros and cons of using VASO to estimate CBV is beyond the scope of this study.

> *We observed a facial valence effect only in the superficial layers of V1, and we interpret this as evidence for feedback to these layers. One alternative explanation for this depth-dependent response profile is related to the widely-characterized superficial bias from draining veins, in which the largest response*

amplitudes are observed in the superficial layers[20]. Even though VASO is thought to mitigate the impact of draining veins[19,21], it is conceivable that BOLD contrast contaminates the VASO measurement to some degree. However, we think this is unlikely for two reasons. First, the VASO responses in our experiment were signal decreases, i.e., negative responses (Fig. 3, but note that the responses were multiplied by -1). This suggests that the removal of the BOLD component of the signal was successful. Second, the layer profile that we report (Fig. 3f) exhibited a clear and prominent decrease at the most superficial cortical depth, rather than a linear increase toward the pial surface, as would be expected from a BOLD layer profile. This observation suggests that the activity profile reflects changes in CBV rather than a vascular confound.

Reviewer #2 (Remarks to the Author):

The authors included a new functional connectivity analysis from the 7T BOLD experiment. The analysis is based on the residuals of the responses for each area in visual cortex and the amygdala.

R2.1.1 Based on figure 2, the correlation coefficient between the visual hierarchy and amygdala (V1, V2 etc etc) appears to be small (black to dark grey color) for the fearful and neutral conditions. The authors compute the difference in connectivity between fearful and neutral condition, reporting that the difference in correlation is significantly different than zero between the amygdala and visual cortex. Difference in connectivity is a derivative measure. I do not have a problem with derivative measures per se, but in this case the starting points (correlation in the fearful and correlation in neutral conditions) are very small (close to zero), indicating in essence no connectivity. We are in presence of two measures that indicate no connectivity, whose difference led to a measure that is interpreted as significant connectivity. I am not entirely convinced by this logic. If we were starting from two significant measures, I would have less problems in taking the difference between the two and interpreting the result.

Response: The reviewer raises an observation that, in retrospect, was not clear in our previous submission. The correlations between the amygdala and visual cortex (V1, V2, etc) are in fact highly significant. The correlations are about R=0.3 on average, well above a stringent statistical threshold, and even after applying a conservative correction for multiple comparisons (Bonferroni).

The correlation values appeared smaller than they actually were in the previous version of the figure because of the grayscale colormap, in which many of the dark colors in the lower third of the range looked uniformly black. We have now used a 'pink' colormap, in which it is easier to visualize the correlation values. Using the pink colormap, it is clear that correlations with the amygdala hover at around 0.3 (see **new Figure 2b-c**, all squares under the 'pink' colormap are significantly above 0, Bonferroni-corrected**)**. We have also included correlation matrices for the fearful, happy, and neutral face conditions in which the numerical correlation values are provided (rather than rely on colorscale) in **new Supplementary Tables 1-3**.

R2.1.2 The observation of low connectivity between V1 (early visual cortex in general) and the amygdala is consistent with the remark made by Reviewer 3 (and the literature).

Response: In our previous response to R3, we note that valence effects in early visual cortex, including V1, have indeed been reported in human EEG/ERP studies[22,23], recordings in awake

monkey[24], and computational modeling[25]. However, fMRI evidence for valence sensitivity in human early visual cortex has been conflicting, with clear effects reported in studies using face stimuli[8,26], and studies using emotional scene and applying decoding analysis[25,27], but not studies using emotional scene and applying univariate analysis[28–30]. We find the seemingly contradictory nature of this prior literature[8,26,27,29,31–34] to be perhaps one of the strongest motivations for our study. We present a large sample of V1 responses to emotional stimuli, measured across multiple imaging modalities (3T, 7T, BOLD, VASO), and in so doing, provide perhaps the strongest support, to date, for the hypothesis that early visual cortex is sensitive to emotional faces.

In our response to R1.2, we note that low connectivity between V1 (and early visual cortex) and the amygdala is expected. This is largely due to a considerably smaller visually-driven response in the amygdala than in V1 in our data, and is consistent with observations in monkeys reported a much lower spike rate in the amygdala compared to visual cortex[6,7], and is consistent with previous fMRI studies showing substantially smaller BOLD responses in amygdala relative to V1[8].

R2.1.3 Are the p values from the Wilcoxon signed rank tests reported in figure 2 corrected for the number of comparisons?

Response: Yes, all statistical values in **Figure 2** are corrected for multiple comparisons using a Bonferroni correction (P x 14 ROIs).

R2.2 Positive Valence effect.
The cortical depth dependent effect for happy faces is now reported (to a limited degree) in the supplementary material. I still believe that a full description of the valence effect for positive faces is necessary to complement the results for fearful faces. For example, it could give insights about the specificity of the cortical depth-dependent results regarding the emotion and the connotation (positive or negative) conveyed.
Moreover, given that the experiment included 3 conditions: fearful, neutral (control) and happy, a reader would naturally expect to see the results from the happy condition as well.

Response: We agree with the reviewer that observations of positive valence effects deserve reporting. We now provide a much fuller reporting of the positive valence effects so readers can easily contrast them with the negative valence effects reported within the same figure. In general, happy faces evoked smaller valence effects than fearful faces. We also now report cortical depth dependent effects for the happy – neutral face contrast in **new Figure 3h**. We have included the widespread positive valence effect (previously Supplementary Figure 4) in **new Figure 1**. We have also included inter-area correlation coefficients for happy face condition and the differences in correlation (happy − neutral) in **new Figure 2c**.

Reviewer #3

R3.1 I believe the authors have revised their work extensively and convincingly, and that this paper will make a solid contribution to the literature. I have 1 issue that could be addressed with minor language revisions.

Response: We thank the reviewer for the positive assessment of the manuscript, and we too hope it will make a solid contribution to the literature.

My only concern is the conflation of face and scene perception in lines 346-351. In this third point, the human studies of scene processing are compared to the current results, under the umbrella concept of valence sensitivity. Please specify which studies used faces, and which used scenes. This is an important difference, as face processing is likely to be supported by distinct mechanisms relative to scenes, as you discuss in the sentences before and after this section, and specifically in lines 465-468.

Response: This is a great suggestion. We agree that the difference in valence sensitivity between face/scene is an important point and are pleased to incorporate this suggestion in the following two paragraphs.

We revised the following paragraph to specify which studies used faces and which used scenes (p. 17, line 367-374).

> *Third, despite converging evidence of valence sensitivity in early visual cortex from human EEG/ERP[22,23], recordings in awake monkey[24], and computational modeling[25], fMRI evidence for valence sensitivity in human early visual cortex has been conflicting, with clear effects reported in studies using face stimuli[8,26], and studies using emotional scene and applying decoding analysis[25,27], but not studies using emotional scene and applying univariate analysis[28–30]. Our results demonstrate clear and reliable valence sensitivity throughout human visual cortex, including in V1.*

We also included a discussion on potentially distinct mechanisms of emotional face and scene processing (pp. 21-22, line 525-537), as nicely pointed out by R3.

> *This may suggest that valence-related feedback signals are stimulus specific, with face stimuli, and perhaps animate objects more generally[35], engaging the circuitry from basal amygdala to V1, and scene stimuli engaging connectivity between ventral visual cortex and V1. Regardless of stimulus type, the valence effect occurs throughout visual cortex in both studies. It is known that face and scene stimuli are associated with distinct patterns of brain activity beyond the amygdala[36]. Two key factors may underlie potentially distinct mechanisms of emotional face and scene processing. First, the heterogeneity in image statistics is smaller across faces than across natural scenes. Second, compared to the direct communicative role of facial expressions, the emotional and social aspects of scene processing are commonly perceived as more indirect and secondary. Thus, future work will need to use network analysis of whole brain dynamics across different imaging modalities to determine whether these widespread valence effects are due to direct influence from the amygdala, feedforward inputs from V1, or a combination of both.*

References cited in this response letter

1. Chen, G. *et al.* Handling Multiplicity in Neuroimaging Through Bayesian Lenses with Multilevel Modeling. *Neuroinformatics* **17**, 515–545 (2019).
2. Chen, G., Taylor, P. A., Cox, R. W. & Pessoa, L. Fighting or embracing multiplicity in neuroimaging? neighborhood leverage versus global calibration. *Neuroimage* **206**, 116320 (2020).
3. Chen, G. *et al.* Beyond linearity in neuroimaging: Capturing nonlinear relationships with application to longitudinal studies. *Neuroimage* **233**, 117891 (2021).
4. Viechtbauer, W. Bias and Efficiency of Meta-Analytic Variance Estimators in the Random-Effects Model: *http://dx.doi.org/10.3102/10769986030003261* **30**, 261–293 (2016).
5. Freeman, J., Donner, T. H. & Heeger, D. J. Inter-area correlations in the ventral visual pathway reflect feature integration. *J. Vis.* **11**, 15–15 (2011).
6. Gothard, K. M., Battaglia, F. P., Erickson, C. A., Spitler, K. M. & Amaral, D. G. Neural responses to facial expression and face identity in the monkey amygdala. *J. Neurophysiol.* **97**, 1671–1683 (2007).
7. Wang, S. *et al.* Neurons in the human amygdala selective for perceived emotion. *Proc. Natl. Acad. Sci. U. S. A.* **111**, (2014).
8. Pessoa, L., McKenna, M., Gutierrez, E. & Ungerleider, L. G. Neural processing of emotional faces requires attention. *Proc. Natl. Acad. Sci. U. S. A.* **99**, 11458–11463 (2002).
9. Boubela, R. N. *et al.* fMRI measurements of amygdala activation are confounded by stimulus correlated signal fluctuation in nearby veins draining distant brain regions. *Sci. Reports 2015 51* **5**, 1–15 (2015).
10. Kanwisher, N., McDermott, J. & Chun, M. M. The fusiform face area: a module in human extrastriate cortex specialized for face perception. *J. Neurosci.* **17**, 4302–11 (1997).
11. Levy, I., Hasson, U., Avidan, G., Hendler, T. & Malach, R. Center–periphery organization of human object areas. *Nat. Neurosci.* **4**, 533–539 (2001).
12. Hasson, U., Levy, I., Behrmann, M., Hendler, T. & Malach, R. Eccentricity bias as an organizing principle for human high-order object areas. *Neuron* **34**, 479–490 (2002).
13. Boynton, G. M., Engel, S. A., Glover, G. H. & Heeger, D. J. Linear systems analysis of functional magnetic resonance imaging in human V1. *J. Neurosci.* **16**, 4207–4221 (1996).
14. Dale, A. M. & Buckner, R. L. Selective averaging of individual trials using fMRI. *Neuroimage* **5**, 329–340 (1997).
15. van Dijk, J. A., Fracasso, A., Petridou, N. & Dumoulin, S. O. Validating Linear Systems Analysis for Laminar fMRI: Temporal Additivity for Stimulus Duration Manipulations. *Brain Topogr.* **34**, 88–101 (2021).
16. van Dijk, J. A., Fracasso, A., Petridou, N. & Dumoulin, S. O. Linear systems analysis for laminar fMRI: Evaluating BOLD amplitude scaling for luminance contrast manipulations. *Sci. Rep.* **10**, 1–15 (2020).
17. Lu, H., Soltysik, D. A., Ward, B. D. & Hyde, J. S. Temporal evolution of the CBV-fMRI signal to rat whisker stimulation of variable duration and intensity: A linearity analysis. *Neuroimage* **26**, 432–440 (2005).
18. Finn, E. S., Huber, L., Jangraw, D. C., Molfese, P. J. & Bandettini, P. A. Layer-dependent activity in human prefrontal cortex during working memory. *Nat. Neurosci.* **22**, 1687–1695 (2019).
19. Huber, L. *et al.* High-Resolution CBV-fMRI Allows Mapping of Laminar Activity and Connectivity of Cortical Input and Output in Human M1. *Neuron* **96**, 1253–1263 (2017).
20. Kay, K. *et al.* A critical assessment of data quality and venous effects in sub-millimeter fMRI. *Neuroimage* **189**, 847–869 (2019).
21. Lu, H., Golay, X., Pekar, J. J. & Van Zijl, P. C. M. Functional magnetic resonance imaging

based on changes in vascular space occupancy. *Magn. Reson. Med.* **50**, 263–274 (2003).
22. Thigpen, N. N., Bartsch, F. & Keil, A. The malleability of emotional perception: Short-term plasticity in retinotopic neurons accompanies the formation of perceptual biases to threat. *J. Exp. Psychol. Gen.* **146**, 464–471 (2017).
23. Keil, A. *et al.* Early modulation of visual perception by emotional arousal: Evidence from steady-state visual evoked brain potentials. *Cogn. Affect. Behav. Neurosci. 2003 33* **3**, 195–206 (2003).
24. Li, Z., Yan, A., Guo, K. & Li, W. Fear-Related Signals in the Primary Visual Cortex. *Curr. Biol.* **29**, 4078-4083.e2 (2019).
25. Kragel, P. A., Reddan, M. C., LaBar, K. S. & Wager, T. D. Emotion schemas are embedded in the human visual system. *Sci. Adv.* **5**, (2019).
26. Koizumi, A. *et al.* Threat anticipation in pulvinar and in superficial layers of primary visual cortex (V1). Evidence from layer-specific ultra-high field 7T fMRI. *eNeuro* **6**, (2019).
27. Bo, K. *et al.* Decoding Neural Representations of Affective Scenes in Retinotopic Visual Cortex. *Cereb. Cortex* **00**, 1–17 (2021).
28. Lang, P. J. *et al.* Emotional arousal and activation of the visual cortex: An fMRI analysis. *Psychophysiology* **35**, 199–210 (1998).
29. Sabatinelli, D., Lang, P. J., Bradley, M. M., Costa, V. D. & Keil, A. The timing of emotional discrimination in human amygdala and ventral visual cortex. *J. Neurosci.* **29**, 14864–14868 (2009).
30. Bradley, M. M. *et al.* Activation of the visual cortex in motivated attention. *Behav. Neurosci.* **117**, 369–380 (2003).
31. Lang, P. J., Fitzsimmons, J. R., Bradley, M. M., Cuthbert, B. N. & Scott, J. Processing emotional pictures: Differential activation in primary visual cortex. *Neuroimage* **3**, S231 (1996).
32. Lang, P. J. *et al.* Emotional arousal and activation of the visual cortex: An fMRI analysis. *Psychophysiology* **35**, 199–210 (1998).
33. Sabatinelli, D. *et al.* Emotional perception: Meta-analyses of face and natural scene processing. *NeuroImage* **54**, 2524–2533 (2011).
34. Bradley, M. M. *et al.* Activation of the visual cortex in motivated attention. *Behav. Neurosci.* **117**, 369–380 (2003).
35. Yang, J., Bellgowan, P. S. F. & Martin, A. Threat, domain-specificity and the human amygdala. *Neuropsychologia* **50**, 2566–2572 (2012).
36. Sabatinelli, D. *et al.* Emotional perception: Meta-analyses of face and natural scene processing. *Neuroimage* **54**, 2524–2533 (2011).

Reviewers' comments:

Reviewer #1 (Remarks to the Author):

The authors addressed all my points. Even though I am still not fully convinced by the small magnitude of the effects, on which the conclusions are based, I do not see anymore any substantial errors and therefore endorse the publication.

Reviewer #2 (Remarks to the Author):

The authors have addressed all of my concerns with the previous version of the manuscript. I think this paper represents a very interesting and solid contribution to the literature.

Below, we provide a point-by-point response to each of the reviewer's comments. The reviewer's comments are indicated by blue font.

Reviewer 1

The authors addressed all my points. Even though I am still not fully convinced by the small magnitude of the effects, on which the conclusions are based, I do not see anymore any substantial errors and therefore endorse the publication.

We thank the reviewer for the careful and thoughtful suggestions on previous rounds of review and for now endorsing publication.

Reviewer 2

The authors have addressed all of my concerns with the previous version of the manuscript. I think this paper represents a very interesting and solid contribution to the literature.

We thank the reviewer for the positive assessment of the manuscript.
